# Substrate-Trapped Interactors of PHD3 and FIH Cluster in Distinct Signaling Pathways

**DOI:** 10.1016/j.celrep.2016.02.043

**Published:** 2016-03-10

**Authors:** Javier Rodriguez, Ruth Pilkington, Amaya Garcia Munoz, Lan K. Nguyen, Nora Rauch, Susan Kennedy, Naser Monsefi, Ana Herrero, Cormac T. Taylor, Alex von Kriegsheim

**Affiliations:** 1 Systems Biology Ireland, University College Dublin, Dublin 4, Ireland; 2 Conway Institute, University College Dublin, Dublin 4, Ireland; 3 Edinburgh Cancer Research Centre, IGMM, University of Edinburgh, Edinburgh EH4 2XR, UK

## Abstract

Amino acid hydroxylation is a post-translational modification that regulates intra- and inter-molecular protein-protein interactions. The modifications are regulated by a family of 2-oxoglutarate- (2OG) dependent enzymes and, although the biochemistry is well understood, until now only a few substrates have been described for these enzymes. Using quantitative interaction proteomics, we screened for substrates of the proline hydroxylase PHD3 and the asparagine hydroxylase FIH, which regulate the HIF-mediated hypoxic response. We were able to identify hundreds of potential substrates. Enrichment analysis revealed that the potential substrates of both hydroxylases cluster in the same pathways but frequently modify different nodes of signaling networks. We confirm that two proteins identified in our screen, MAPK6 (Erk3) and RIPK4, are indeed hydroxylated in a FIH- or PHD3-dependent mechanism. We further determined that FIH-dependent hydroxylation regulates RIPK4-dependent Wnt signaling, and that PHD3-dependent hydroxylation of MAPK6 protects the protein from proteasomal degradation.

## Highlights

• The proteomic screen for FIH and PHD3 substrates identifies numerous interactors
• Potential substrates are enriched in numerous hypoxia-regulated pathways
• FIH regulates RIPK4 kinase activity by direct hydroxylation
• Hydroxylation of Pro25 by PHD3 regulates MAPK6 (Erk3) protein stability

## Graphical Abstract

**Figure:**
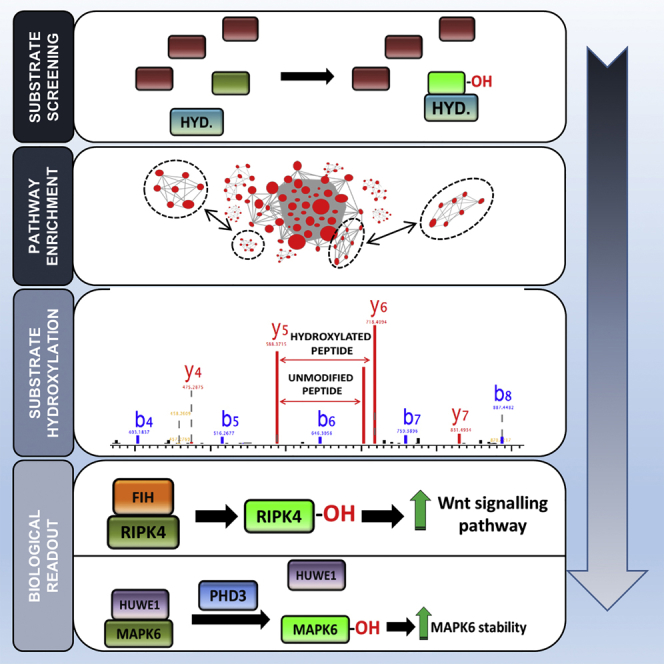

## Teaser

Using quantitative interaction proteomics, Rodriguez et al. identify numerous potential hydroxylase substrates clustering in hypoxia regulated pathways and show that hydroxylation of two of these substrates, MAPK6 (Erk3) and RIPK4, has consequences for cellular functions.

## Introduction

Post-translational modifications (PTMs) of proteins provide versatile mechanisms to regulate protein activity and protein interactions. The aliphatic side-chains of lysine, asparagines, aspartic acid, tryptophan, and proline as well as methylated lysines and arginines can all be hydroxylated in an oxygen and 2-oxo glutarate- (2OG) dependent mechanism by a family of enzymes termed the (2OG)-oxygenases (Loenarz and Schofield, 2008, Winston et al., 1999).

Initial observations that (2OG)-oxygenases can post-translationally modify proteins came from studies involving collagen and related proteins in which multiple proline and lysine residues were found to be hydroxylated. Subsequently, it was discovered that hydroxylation could regulate functions and degradation of HIF1α (Ivan et al., 2001, Jaakkola et al., 2001). Upon hydroxylation and binding of VHL, HIF1α is poly-ubiquitinated and targeted for degradation by the proteasome. A third hydroxylation on a C-terminal asparagine reduces the transcriptional activity of the complex (Hewitson et al., 2002).

It has become obvious that hypoxia and hydroxylases regulate many aspects of the cellular signaling machinery, but, despite high interest in detecting novel substrates, progress has been slow, especially with respect to the HIF hydroxylases PHD1, PHD2, and PHD3. So far a few experimental strategies proved successful in detecting novel substrates. Mass spectrometry based proteomics was used successfully for FIH (Cockman et al., 2009) and yeast 2-hybrid screens identified some potential PHD substrates (Köditz et al., 2007). Several additional PHD substrates were identified by screening for the proposed consensus sequence LxxLAP (Luo et al., 2011, Moser et al., 2013). However, only a relatively small number of PHD substrates were successfully identified to date, and we still lack full understanding of how hydroxylation affects signaling pathways beyond the canonical HIF-pathway.

To address these questions, we employed an unbiased, quantitative mass-spectrometry-based approach to detect PHD3 and FIH substrates, based on a pharmacological substrate-trap strategy which was previously used for detecting multiple new and confirming several known FIH substrates (Cockman et al., 2009). PHD3 was selected because it is expressed both in the nucleus and in the cytoplasm. This ubiquitous distribution contrasts with the nuclear expression of PHD1 and the predominantly cytoplasmic localization of PHD2 (Metzen et al., 2003). We expected that a broader distribution pattern of PHD3 would result in a larger substrate pool.

## Results

**Figure 1:**
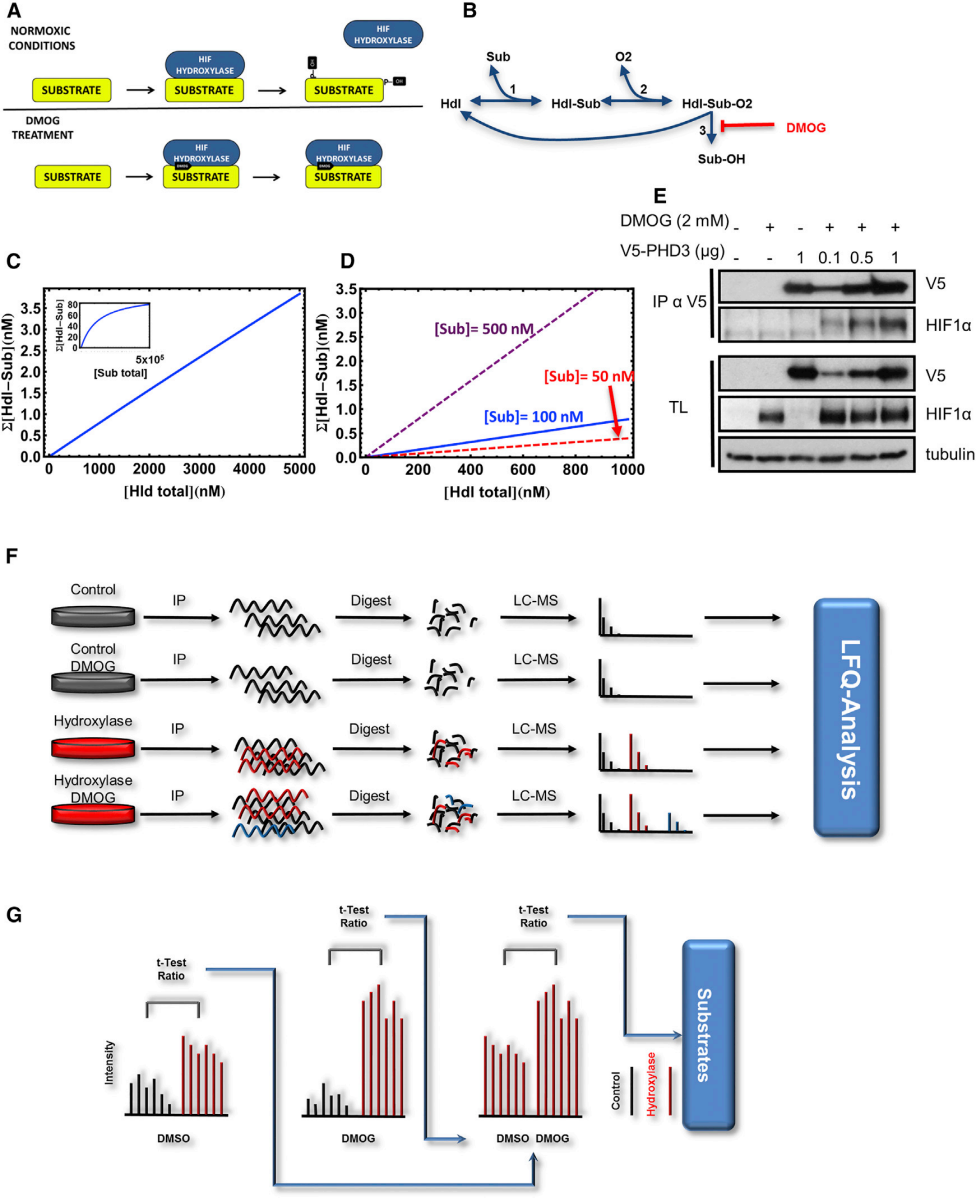
Steady-State Model of Hydroxylase Substrate-Trap and Experimental Design of Hydroxylase-Substrate Screen (A) Cartoon of how the substrate-trap functions. In the absence of DMOG, the hydroxylases bind to the substrate and are released upon its hydroxylation. In the presence of DMOG, the hydroxylation is inhibited and the enzyme-substrate complex is trapped. (B) Reaction scheme of a steady-state model for hydroxylase-substrate interaction under inhibitor (DMOG) treatment. The details of the model with equations are given in the Supplemental Information. (C) Dependence of total hydroxylase-substrate (Hdl-Sub) binding in response to gradual overexpression of the hydroxylase (Hdl) enzyme, showing a robust linear dependence over a wide dynamic range of the enzyme concentration. The inbox figure shows saturation appearing only at extremely high enzyme concentration. (D) Dependence of total substrate-hydroxylase (Hdl-Sub) binding in response to gradual overexpression of the hydroxylase (Hdl) enzyme under varying substrate concentration. A linear dependence is still robustly observed for low and high substrate levels. (E) Validation of the model. V5-PHD3 or an empty vector was transfected at the indicated amounts into HEK293T cells. At 24 hr post-transfections, the cells were treated with 2 mM DMOG for 3 hr. The cells were lysed, PHD3 immunoprecipitated, and proteins were separated by PAGE, electro-blotted, and detected by the indicated antibodies. (F) Schematic illustration of the mass spectrometry based hydroxylase screen. The HEK293T cells were transfected with the tagged hydroxylases and treated with DMOG. The hydroxylases and their binding proteins were immunoprecipitated, digested, and analyzed by mass spectrometry. The proteins were identified and subsequently quantified by LFQ. (G) Illustration of data analysis. The LFQ intensity values were averaged and filtered via a t test and ratio cutoff versus the respective negative controls. All significant hits were then additionally compared to each other after the hydroxylase input was normalized. The proteins whose bindings were significantly increased by DMOG were deemed to be potential substrates.

Dimethyloxaloylglycine (DMOG) “traps” the hydroxylase enzyme-substrate complex in an inactive state (Cockman et al., 2009). Whereas a 2OG-bound complex releases the product upon hydroxylation, the reaction and product release are inhibited if DMOG is bound (Figure 1A). Therefore, the presence of DMOG in the cell not only inhibits the accumulation of hydroxylated proteins, but also increases the amount of substrate bound to the hydroxylase.

In order to determine whether overexpression of a hydroxylase affects the enzyme-substrate complex formation under DMOG treatment, we developed a mathematical steady-state model of the interaction based on the reaction steps leading to hydroxylation of HIF1α by prolyl-hydroxylases (Rose et al., 2011) (Figure 1B; Supplemental Information). The total DMOG-stabilized substrate-hydroxylase complex, in response to increasing concentrations of the hydroxylase, shows a linear relationship over several orders of magnitude (Figure 1C). This linear relationship persists even when substrate levels (Figure 1D) or the binding affinities vary strongly (Figures S1A and S1B). In order to confirm this prediction, we transfected HEK293T cells with increasing amounts of V5-tagged PHD3 and treated them with DMOG. We immunoprecipitated PHD3 and analyzed the amount of endogenous HIF1α, a low to medium abundant transcription factor, bound to PHD3. In agreement with the mathematical model, increasing amounts of cellular PHD3 co-immunoprecipitated and bound increasing amounts of HIF1α, which was at a constant concentration in the cells (Figure 1E). In conclusion, overexpression of the hydroxylase was not likely to saturate the complex formation for low, medium, and highly abundant substrates, allowing us to express tagged hydroxylases as baits for the substrate screen.

To screen for substrates, we selected HEK293T as cell line models as it maintains a transfection efficiency of above 99% even when transfecting low amounts of DNA, thus, we would be able to titer the transient overexpression close to the physiological range (Figures S1C–S1G). The cells were transfected with either a V5-tagged hydroxylase or an empty vector. Overexpression of FIH and PHD3 was determined to be 10- and 30-fold over the endogenous, normoxic level, respectively (Figures S1H and S1I). Given that cellular levels of low abundant proteins can vary by an order of magnitude within an isogenic cell line (Yuan et al., 2011) and that PHD3 can be induced several 10-folds in chronic hypoxia (Appelhoff et al., 2004), these levels of overexpression were not beyond the expected physiological range. Subsequently, the precipitated proteins were identified and quantified by label-free quantification, as implemented in MaxQuant (MaxLFQ) (Tate et al., 2013) (Figure 1F). MaxLFQ has a performance comparable to isotope-based labeling methods when it comes to detecting relative changes in protein abundance (Cox et al., 2014). In addition, intensity values determined by MaxQuant retain information about the relative abundance of distinct proteins within a complex, albeit at lower accuracy (Fabre et al., 2014). Thus, MaxLFQ intensities not only accurately represent changes in protein interaction, but can also be used to rank the proteins in terms of likely relative abundance. We extracted the specific interactome by comparing the V5-hydroxylase, DMOG, and untreated, sample versus their corresponding controls. Next, we extracted the DMOG-induced specific fraction by comparing intensities of the specific interactome between the untreated and DMOG treated V5-hydroxylase samples (Figure 1G). This fraction should be enriched for trapped substrates, proteins which specifically associate with the hydroxylases and are induced by DMOG, but are not necessarily substrates, and proteins which are in complex with a substrate, but do not bind to the hydroxylases directly.

### Identification of DMOG-Induced Hydroxylase Interactome

**Figure 2:**
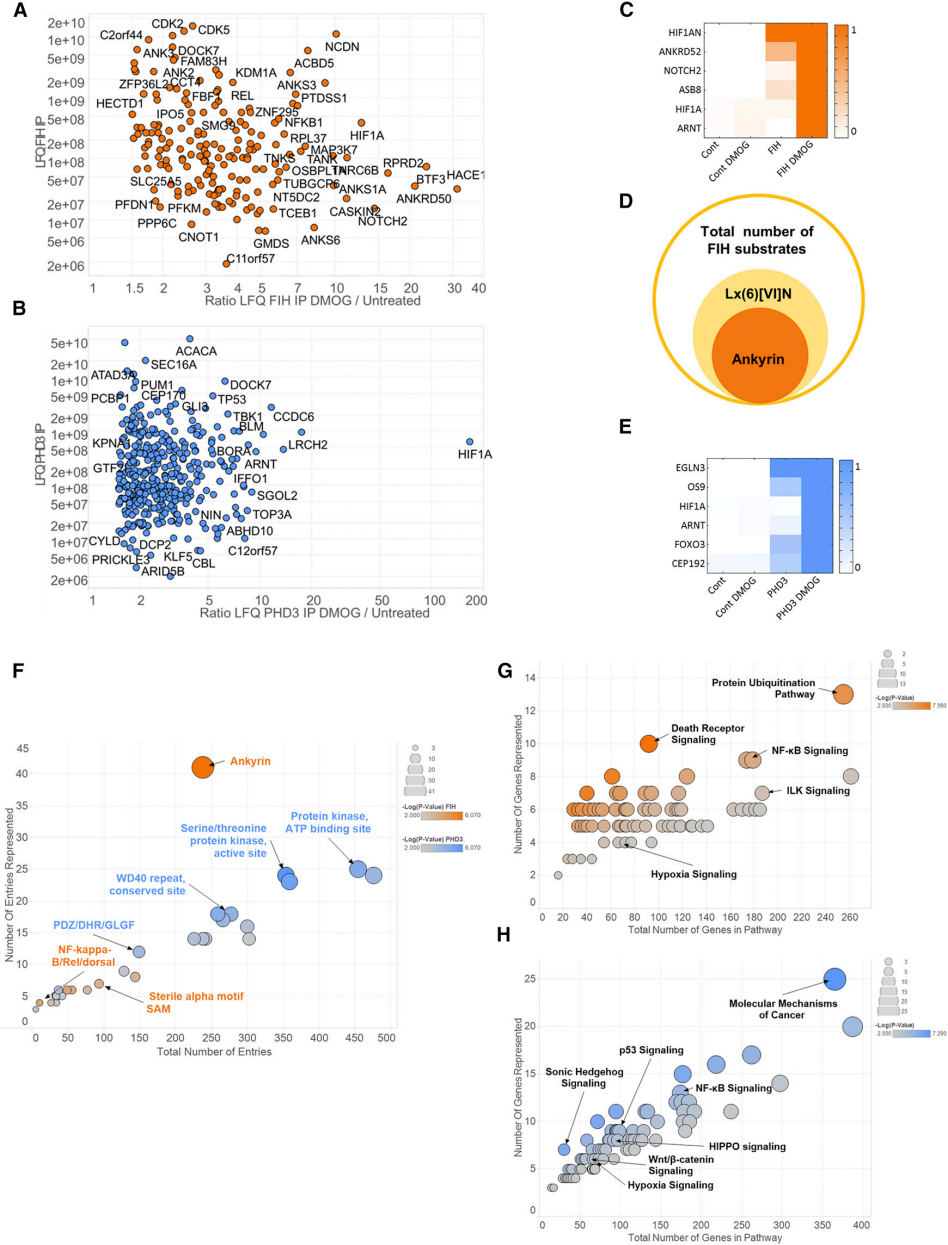
Overview of FIH and PHD3 DMOG-Trapped Interactors (A) Scatterplot of LFQ-intensities over DMOG/untreated ratio of 192 proteins specifically binding to FIH upon DMOG treatment. The selected interactors were labeled with gene name. (B) Scatterplot of LFQ-intensities over DMOG/untreated ratio of 388 proteins specifically binding to PHD3 upon DMOG treatment. The selected interactors were labeled with gene name. (C) Normalized LFQ-intensities of FIH (HIF1AN) and selected, known substrates. The heatmap representation of normalized LFQ-intensity values as obtained from FIH immunoprecipitations and sorted in descending order by intensity is shown. (D) Venn diagram of DMOG-trapped FIH interactors containing an FIH consensus motif or AR. (E) Normalized LFQ-intensities of PHD3 (EGLN3) and selected, known substrates and interactors. The heatmap representation of normalized LFQ-intensity values as obtained from FIH immunoprecipitations is shown. (F) Graphical representation of protein domains enriched in either the FIH (orange) or PHD3 (blue) substrate screen. The cutoff is a Benjamini Hochberg corrected p value of 0.01. (G) Graphical representation of pathways enriched in the FIH substrate screen. The cutoff is a p value of 0.01. (H) Graphical representation of pathways enriched in the PHD3 substrate screen. The cutoff is a p value of 0.01.

In the combined FIH and PHD3 searches, we were able to detect and quantify over 3,000 proteins, most of them being non-specific binders. Among the specific interactions, as judged by a t test and ratio cutoff of p < 0.05 and ratio >2-fold, DMOG induced the association of 192 proteins with FIH (Table S1) (Figures 2A and S2A) and 388 with PHD3 (Table S2) (Figures 2B and S2B). To test the sensitivity of our method, we screened the predicted FIH substrates for bona fide known substrates. We readily identified HIF1α, NOTCH2, TNKS2, NFKBIB, and several Ankyrin-repeat (AR) proteins (Figure 2C). We did not detect HIF2α, as it is not expressed in HEK293T cells (Nguyen et al., 2013). In addition, an InterPro search revealed that 41 of the 192 proteins contained ARs. Furthermore, we screened the DMOG-induced FIH interactors for the FIH-consensus sequence Lx(6)[VI]N, detecting 80 proteins containing the sequence at least once. Assuming that true FIH substrates contain ARs, or at least the FIH consensus sequence, our screen has a specificity to detect FIH substrates between 21% (based on AR) and 41% (based on the consensus sequence) (Figure 2D).

It is not surprising that only a subset of the DMOG-trapped proteins are substrates, as mass spectrometry (MS) based interaction proteomics inherently identifies entire complexes rather than binary interactions. Specifically, we detected HIF1β (ARNT), which is likely to be indirectly bound to FIH by forming a hetero-dimer with directly bound HIF1α. Although the dynamic profile of HIF1β association with FIH is similar to HIF1α, the directly binding protein is present at a higher LFQ intensity. Therefore, of two proteins, which are in a tight complex and have similar dynamic interaction profiles, the more abundant one is more likely to be the directly bound partner. This trend should allow triaging the candidates for further validation. In order to reveal indirect interactors, we uploaded the list of potential substrates into the STRING database (http://www.string-db.org) and limited the links between nodes to experimentally validated protein-protein interactions within the FIH (Figure S3A) or PHD3 (Figure S3E) network. We decided to test the predictive power of our triage on the TCEB1 and TCEB2 complex, as both proteins are connected to other potential FIH substrates (Figure S3A). When we plotted the four most abundant proteins within the cluster, we saw that ASB8 was the most abundant protein, followed by TCEB1, TCEB2, and HIF1A (Figure S3B). When we plotted the DMOG-induced increase of interaction with FIH, a measure of the dynamic interaction profile, we saw that ASB8, TCEB1, and 2 had similar values of induction (between 4- and 6-fold), whereas the interaction with HIF1A was induced 12-fold (Figure S3C). Overall, these data suggested that HIF1A and TCEB1/TCEB2/ASB8 were predominantly in different complexes, with HIF1A and ASB8 the likely substrates. We were able to confirm that ASB8 was hydroxylated on N80 (Figure S3D), whereas we were not able to detect any hydroxylated asparagine residue in TCEB1 or TCEB2, despite expressing FIH and identifying unmodified counterpart peptide.

We subsequently screened the PHD3 interactome data for proteins which have been shown to be hydroxylated by PHDs (see Figure 2E). As with FIH, we readily detected HIF1α and β. In addition, we found CEP192, a centrosomal protein, which has been recently described to be hydroxylated by PHD1 (Moser et al., 2013). We also identified LIMD1 (Foxler et al., 2012) and OS9 (Baek et al., 2005) as specific DMOG-induced proteins. Both proteins have been described to interact with PHDs and HIF, although they have not been identified as PHD substrates. The fact that both proteins can bind PHD3 in normoxia, albeit at lower levels, suggests that they can bind PHD3 independently of HIF, which is absent under normoxic conditions. Additionally, we detected FOXO3a and DYRK1, two recently discovered PHD1 substrates, as a specific and DMOG-induced interactor (Lee et al., 2016, Zheng et al., 2014). We failed to identify two PHD3 substrates, PKM2 (Luo et al., 2011) and TELO2 (Xie et al., 2012). Both proteins were detected in our unfiltered screen, but PKM2 was not deemed to be a specific PHD3 interacting protein, as it was present with equal intensity in the negative controls. On the other hand, TELO2 was identified as a PHD3 interacting protein under untreated and DMOG conditions, but was not assigned as a substrate because the interaction was diminished by DMOG. PKM2 is very highly expressed and appears to bind to agarose beads in an unspecific fashion, thus masking the interaction with PHD3. TELO2 on the other hand, may bind to PHD3 via several mechanisms, of which one could be enhanced by DMOG-inhibited PTMs, would these be hydroxylations or other modifications. Such a mechanism makes biological sense as it would induce switch-like hydroxylation of TELO2 in response to a graded oxygen input; however, this is purely speculation and future experiments will have to test this hypothesis.

In addition, to determine protein changes induced by a 4 hr DMOG treatment, we quantified the expression of 8,000 protein groups by mass spectrometry. We matched this information with the interaction data to identify proteins whose altered association with the hydroxylases may be a result of expression changes rather than changes in the affinity. Surprisingly, although the protein expression of several interactors was altered, these changes were generally less pronounced. Overall, only the expression of HIF1A, GLI3, GLI2, CDC20, and NFKBIE was greater than the DMOG-dependent induction observed at the interactome, which indicates that only these proteins are candidates for induced interactors, which may not be necessarily substrates.

FIH has been previously shown to bind and hydroxylate asparagines in AR, and we hypothesized that PHD3 may also have a preference for specific protein domains. Thus, we determined which protein domains were enriched in the FIH and PHD3 substrate data set. As expected, AR were highly enriched in the FIH set and stood out when compared to the additional domains enriched in the set (Figure 2F, orange). In contrast, no single protein domain was predominantly enriched in the PHD3 substrate set. Protein kinases, WD40 and PDZ-domain proteins were significantly enriched (Figure 2F, blue), but given the absence of a clear outlier, such as AR for the FIH substrates, we have to conclude that PHD3 does not preferentially interact with any individual protein domain.

### Pathway-Centered Analysis of the DMOG-Dependent Interactome

Hypoxia and hydroxylases have been shown to regulate several signaling pathways outside the canonical HIF network (Lenihan and Taylor, 2013, Moser et al., 2013, Xie et al., 2012). Enrichment of such pathways would provide additional confirmation that we have identified bona fide substrates. We mapped our data on pathway databases using Ingenuity Pathway Analysis (IPA). Figure 2G gives an overview of the pathways enriched in the FIH substrate screen. Aside from the HIF pathway, the NFκB, and ubiquitination signaling networks were heavily overrepresented in the sample set. Next, we analyzed the PHD3 substrate data, and we detected that the HIF pathway was enriched (Figure 2H). In addition, signaling pathways related to cancer, including NFκB, Hedgehog, p53, Wnt, and Hippo were significantly overrepresented. Reassuringly, all these pathways have been shown to be regulated by hypoxia, and in the case of NFκB and p53 pathways, some effectors also have been shown to be hydroxylated by either PHDs or FIH (Cockman et al., 2006, Cummins et al., 2006, Janke et al., 2013, Scholz et al., 2013, Xie et al., 2012).

**Figure 3:**
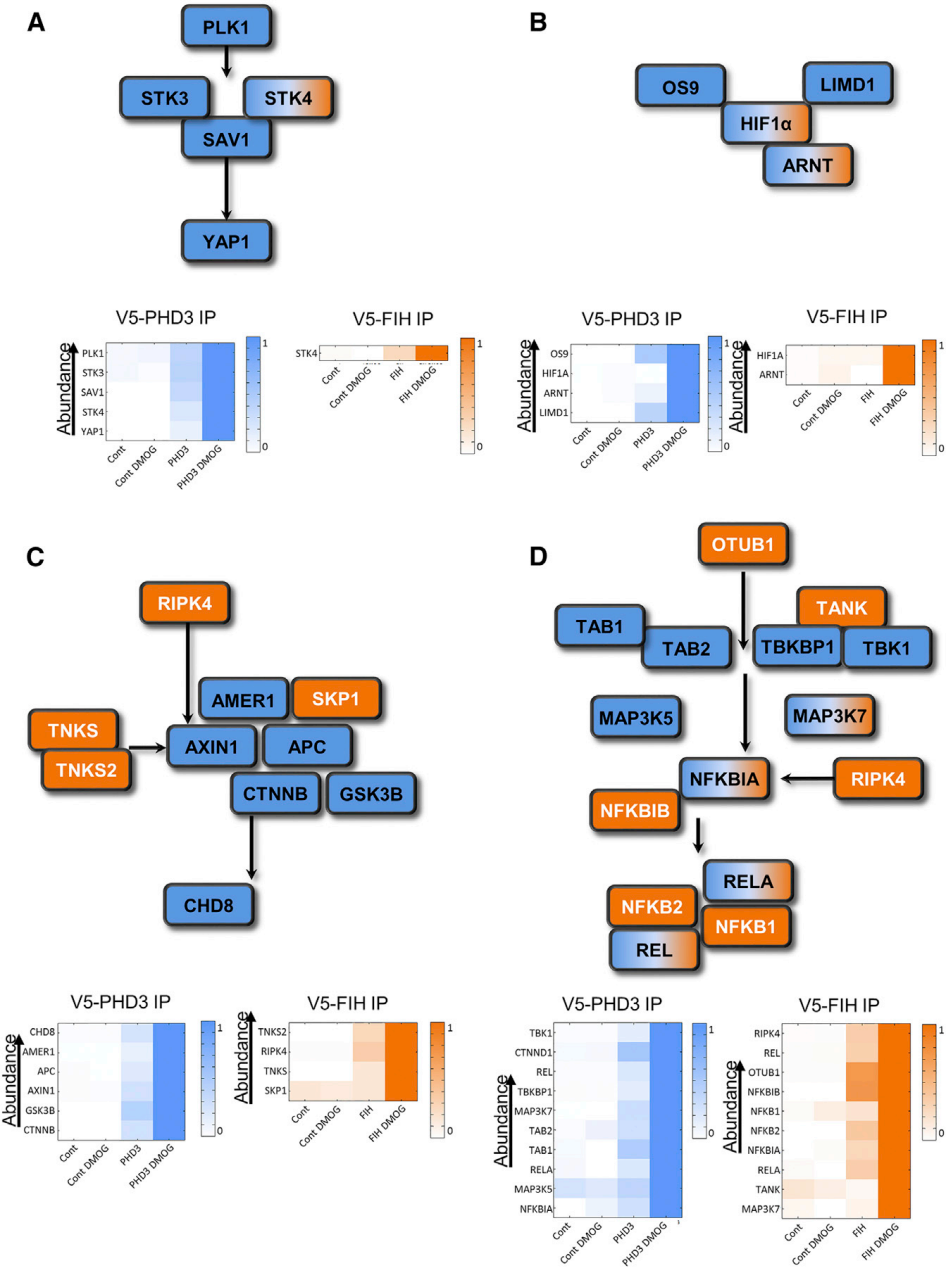
Schematic Illustration of Pathways Enriched in the Substrate Screen Potential PHD3 substrates are shown as blue, FIH substrates as orange, and proteins which bind both FIH and PHD3 in a DMOG inducible way are show as blue/orange boxes. The heatmaps represent the normalized LFQ-intensities of the PHD3 IP (blue) or FIH IP set (orange) and sorted in descending order by intensity. (A–C) Members of the core Hippo-pathway are trapped by PHD3/FIH and DMOG. The core MST1/MST2/Salvador (STK4, STK3, and SAV1) complex interacts with PHD3 in a DMOG-inducible fashion. In addition, the upstream activator PLK1 and the downstream effector YAP1 behave in an analogous manner. In addition, the MST1/FIH interaction is also induced by DMOG (B) FIH and PHD3 interact in a DMOG-dependent manner with core members of the HIF1α-pathway HIF1α/β, OS9, LIMD1 (C) PHD3 interacts in a DMOG- dependent manner with core members of the β-catenin degradation complex. FIH may regulate the upstream kinase RIPK4 and the ADP-Ribosylases TNKS1/2. (D) Schematic illustration of members of the wider NFκB pathway identified in the substrate screen.

Interestingly, these results also indicate that hydroxylation may simultaneously affect several proteins in a pathway in distinct complexes. To corroborate this observation, we systematically mapped proteins identified in the substrate screen onto known signaling pathways. There are four examples that are shown in Figure 3. A substantial proportion (∼50) of potential substrates were bound to FIH as well as PHD3, suggesting that both hydroxylases not only cross-regulate pathways, but also frequently co-regulate individual pathway nodes as seen for HIF1α.

We frequently observed that proteins, which are part of the same multiprotein complex, appear to co-purify with the hydroxylases. For example STK4, STK3, and their scaffold SAV associated with PHD3 in a substrate-like manner (Figure 3A). Given that these proteins form a tight complex (Hauri et al., 2013), it is plausible that only one protein is directly bound to PHD3, most likely STK3 due to the interaction abundance and profile. Within the wider HIF-pathway we identified the HIF-heterodimer, we also detected OS9 and LIMD1 as DMOG induced PHD3 interactors (Figure 3B). The intensity distribution showed that both proteins could interact with PHD3 independently of HIF, as we detected both proteins specifically interacting with PHD3 in the absence of HIF1α in the untreated data set. In the Wnt-pathway, a large proportion of the β-catenin degradation complex associated with PHD3 (Figure 3C). Additionally, the AR proteins RIPK4, TNKS, and TNKS2 bound to FIH in a DMOG-dependent fashion, as did the ubiquitin-ligase SKP1. Most potential substrates were matched to the NFκB-pathway (Figure 3D). This observation ties in with a wealth of data, which has demonstrated that hypoxia regulates this pathway at multiple levels in a PHD and FIH-dependent manner (Cockman et al., 2006, Cummins et al., 2006, Scholz et al., 2013, Shin et al., 2009, van Uden et al., 2011). The number of potential substrates in distinct protein complexes supports the idea that the pathway is not regulated by a single master controller, but rather by distributed control.

In summary, the pathway analysis suggests that hydroxylation controls whole regulatory programs rather than single network nodes and hence may serve to coordinate signaling pathways in a highly integrated fashion. Nevertheless, as the pathway analysis has been performed on the entire DMOG-trapped interactome, the enrichment does not necessarily represent a direct degree of regulation. Due to the trapping of protein complexes, some pathways may have been overrepresented.

### Confirmation of RIPK4 and MAPK6 as Substrates

To prove that a protein is indeed a substrate requires the identification and quantitation of the hydroxylation sites in the presence and absence of hydroxylase activity. If the protein is hydroxylated, the question arises how it affects the molecular and biological function of the target. The majority of hydroxylations have been shown to alter protein-protein binding and identifying hydroxylation-dependent changes in the interactome should give an indication as to what interactions are regulated by the modification. Therefore, we designed a screen which allowed us not only to quantify the hydroxylation status, but also to quantify the interactome of the selected target.

We decided to confirm if two proteins were indeed substrates, one for either hydroxylase analyzed in our screen (Figures S3A and S3E). As selection criteria, we limited the list of prospective substrates to those which were the most intense hydroxylase interactors detected within a co-precipitated complex.

We elected to focus on RIPK4 as a potential FIH-substrate (Figure S4A). RIPK4 is a receptor bound kinase (Bertrand et al., 2011, Meylan et al., 2002), which has not yet been found to interact with any of the other predicted FIH substrates. RIPK4 regulates the Wnt pathway and has been recently shown to stabilize β-catenin by phosphorylating Dishevelled (Huang et al., 2013). Moreover, RIPK4 has been shown to regulate the NFκB pathway by affecting the upstream signaling by binding to TRAF proteins (Meylan et al., 2002).

**Figure 4:**
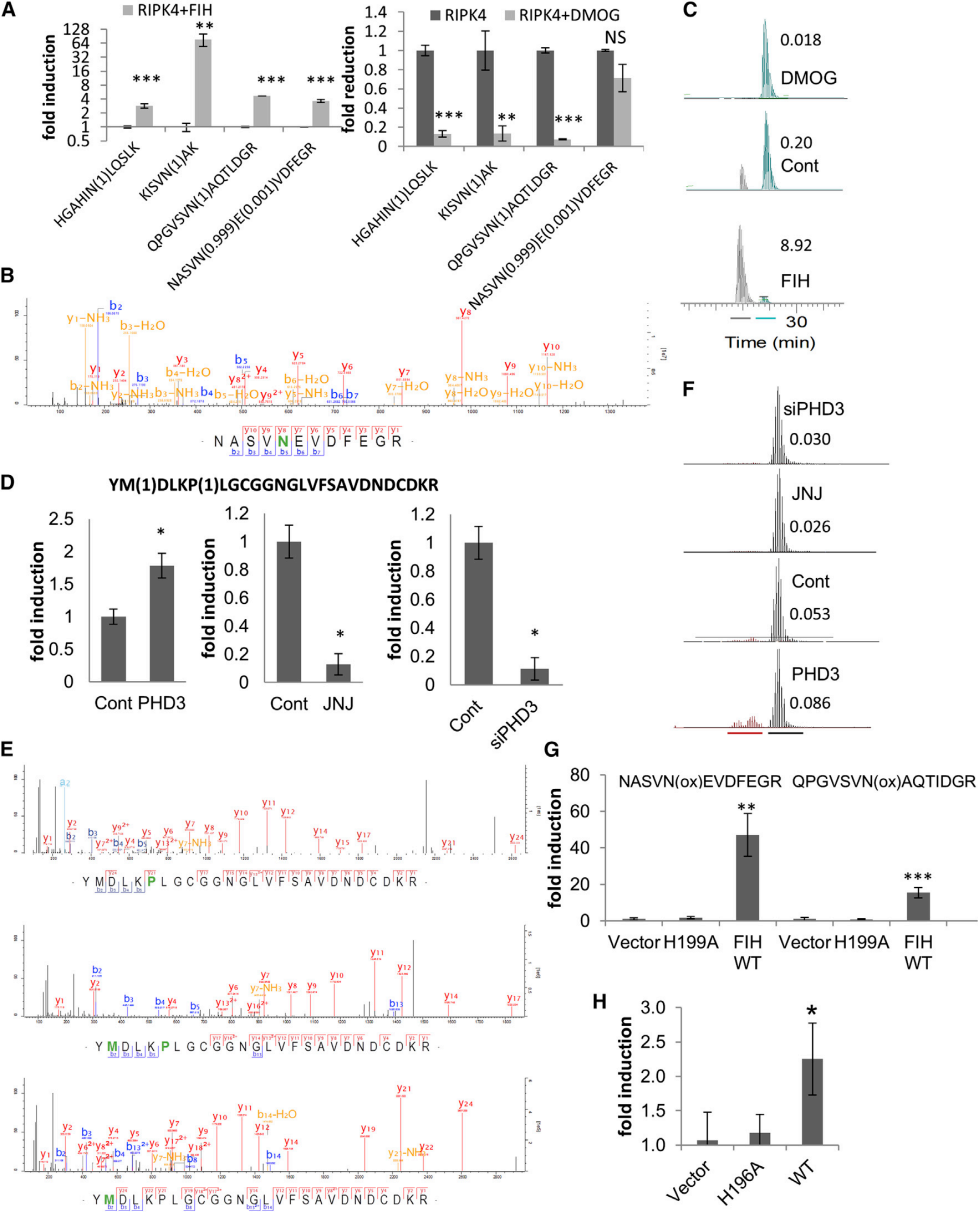
Mass-Spectrometry-Based Targeted Hydroxylation Site Screen (A) Bar graph represents the normalized hydroxylation ratio of RIPK4 peptides in the presence/absence of DMOG or overexpressed FIH. The identified peptides are on the x axis, with the localization probability of the hydroxylation in brackets. The error bars represent SEM and n = 3. (B and C) Representative fragmentation spectrum of hydroxylated NASVN(ox)EVDFEGR (C) XIC of NASVN(ox)EVDFEGR (gray) and non-hydroxylated NASVNEVDFEGR (petrol). The numbers represent the ratio of hydroxylated over the corresponding non-hydroxylated peptide. (D) Bar graph represents the normalized hydroxylation ratio of MAPK6 peptides YM(ox)DLKP(ox)LGCGGNGLVFSAVDNDCDKR over YM(ox)DLKPLGCGGNGLVFSAVDNDCDKR in the presence/absence of JNJ or overexpressed V5-PHD3 or PHD3 specific siRNA. The error bars represent SEM and n = 3. (E and F) Representative fragmentation spectra of P(ox), M(ox), and P(ox)M(ox) of MAPK6 peptide (F) XIC of doubly hydroxylated/oxidized (burgundy) and M(ox) (black) MAPK6 peptide. The numbers represent the ratio of doubly hydroxylated/oxidized over the corresponding M(ox) peptide. (G) Bar graph represents the normalized hydroxylation ratio of RIPK4 peptides following an in vitro hydroxylation assay in the presence of HEK293T lysate expressing a vector control, H199A, or WT V5-FIH. The error bars represent SEM and n = 2. (H) Bar graph represents the normalized hydroxylation ratio of one MAPK6 peptide following an in vitro hydroxylation assay in the presence of HEK293T lysate expressing a vector control, H196A, or WT V5-PHD3. The error bars represent SEM and n = 2.

We quantified the hydroxylation and normalized the value by dividing the intensity of the hydroxylated peptide by the intensity of the unmodified, corresponding peptide. Subsequently, we determined which sites were statistically different between the FIH-overexpressing and the DMOG-treated samples and were present at higher levels in the FIH-overexpressing sample. There were four peptides that fulfilled these conditions, all containing a hydroxylated asparagine (Figures 4A–4C and S4C–S4E) matching the general consensus sequence for FIH, L(x_6_)ΨN.

We selected MAPK6 as a potential PHD3 target (Figure S4B). As with RIPK4, MAPK6 has not been shown to be regulated by hydroxylation or hypoxia. Our interest in MAPK6 was heightened by the technical challenge of detecting a hydroxylation site on a protein which is continuously degraded by the proteasome (Coulombe et al., 2003), a trait that MAPK6 shares with HIF1α.

We transfected C-terminally FLAG-tagged MAPK6 with or without V5-PHD3, incubated with a PHD-specific inhibitor JNJ-42041935 (JNJ) (Barrett et al., 2011) or transfected Control of PHD3 specific small interfering RNA (siRNA) in the presence of the proteasome inhibitor MG132 to limit the plausible effects of PHD3 on MAPK6 protein stability. We analyzed the data as above and detected several hydroxylation sites of which only Pro25 hydroxylation was significantly altered (Figures 4D–4F and S5). In the same peptide, we detected an additional oxidation of the methionine. The oxidation of the methionine decreased the hydrophobicity of the peptide more than the proline hydroxylation, resulting in a shift in the elution time, which allowed us to completely resolve elution profiles for both isobaric peptides. This enabled us to calculate the ratios for the P(ox)/non-modified (Figure S5) and the P(ox)M(ox)/M(ox) (Figures 4D and 4E) independently of each other. The identified site (YMDLKP(ox)LGCGG) does not match the LxxLAP motif, but matched a more degenerated, ΦxxLxP, motif.

To establish whether the asparagine and proline residues detected could be hydroxylated in vitro, we incubated biotin tagged 21 amino acids long peptides surrounding either Asp(646) (LLAKQPGVSV**N**AQTLDGRTPL) or Pro(25) (DLGSRYMDLK**P**LGCGGNGLVF). We incubated the peptides with lysates of HEK293T cells overexpressing V5-FIH, V5-PHD3, or a vector. We readily detected an oxidized peptide in the samples, although closer inspection of the fragmentation spectra revealed that all the peptides were oxidized exclusively on the biotin residue. Consequently, we attempted a second in vitro assay, where we used the in vitro translated (IVT) full-length proteins as substrates instead of the purified peptides. As before, we incubated the purified proteins with lysates of HEK293T cells overexpressing wild-type (WT) V5-FIH, WT V5-PHD3, their respective inactive mutants or a vector. This time we were able to detect two of the four peptides hydroxylated on the asparagine residue (Figure 4G). The basal hydroxylation efficiency of the lysates from the vector and H199A FIH mutant transfected cells were very low and hydroxylated peptides were hardly detectable. In contrast, asparagine hydroxylations could be easily observed and quantified in in vitro assay containing overexpressed V5-FIH, resulting in a 20- to 50-fold induction of asparagine hydroxylation. Disappointingly, we failed to detect two hydroxylated asparagine residues which were detected in the cellular assay. Nevertheless, given the strong data obtained from the cellular assays, in terms of quantification of the hydroxylation, localization in the AR and the matching consensus sequence, we must conclude that for unknown reasons the in vitro assay is giving us false negatives.

In addition, we were also able to detect and quantify the proline hydroxylation in the IVT-MAPK6. Pro(25) which was increased 2-fold in the V5-PHD3 sample in comparison to the vector and H196A PHD3 control (Figure 4H).

### Biological Consequence of FIH-Dependent Hydroxylation of RIPK4

The inclusion of a vector control in the hydroxylation/interactome screen allowed us also to identify proteins which specifically interact with RIPK4 and MAPK6, as well as to determine whether blocking the hydroxylation alters the stoichiometry of the interaction. It is generally accepted that most proteins function as part of multiprotein complexes. Therefore, hydroxylation-dependent changes in the interactome should provide an indication of how hydroxylases shape the signaling of these substrates. After comparing the LFQ-intensities of the RIPK4 and MAPK6 immunoprecipitations to their respective negative controls, we isolated 333 interactors for RIPK4 (Table S3) and 276 interactors for MAPK6 (Table S4).

**Figure 5:**
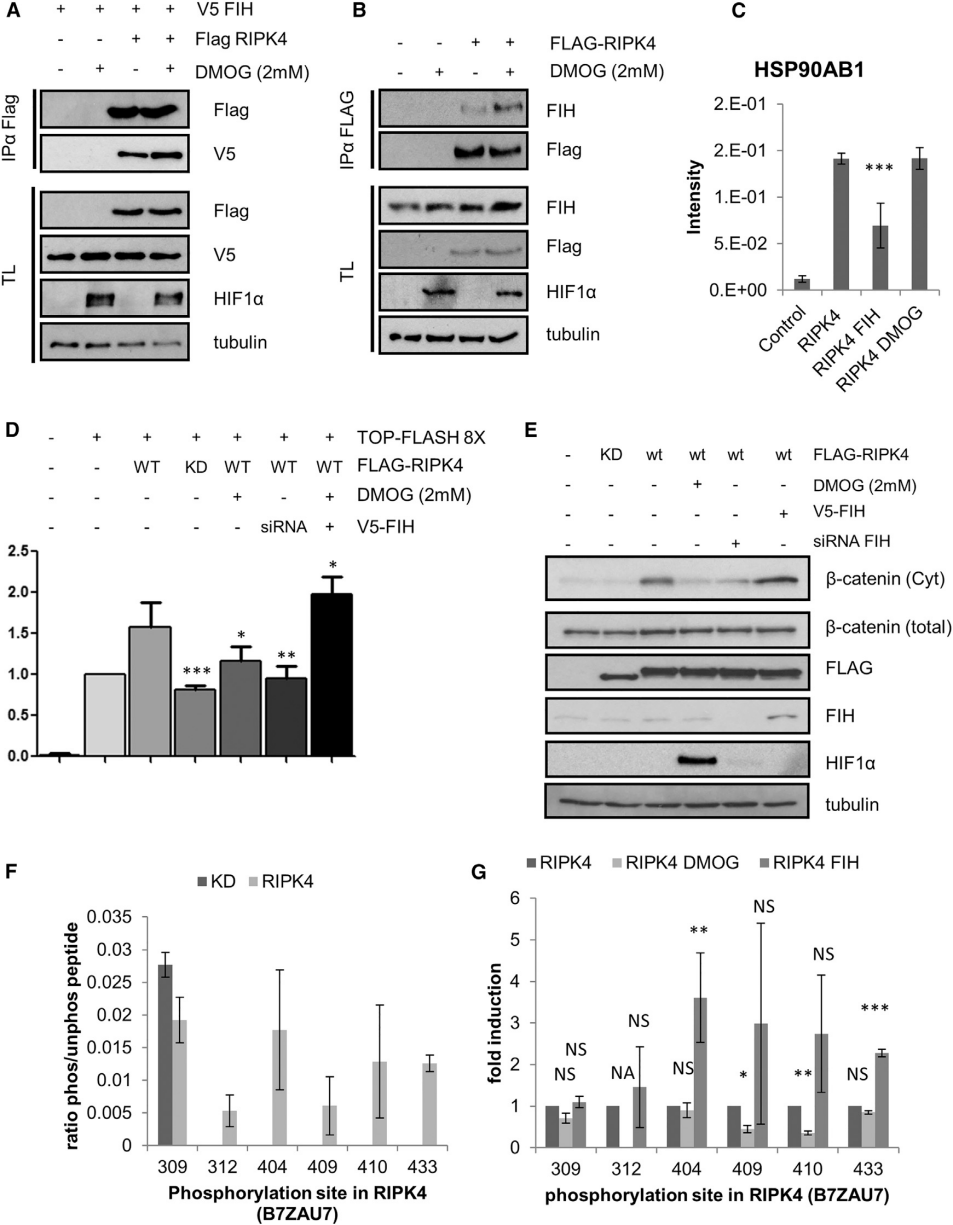
FIH Potentiates RIPK4 Signaling (A) FIH interacts with RIPK4 in a DMOG-dependent fashion. The HEK293T cells were transfected with FLAG-tagged RIPK4 and with V5-FIH as indicated and treated for 4 hr with DMOG 24 hr post transfection. The cells were lysed, FLAG-RIPK4 immunoprecipitated, and proteins were separated by PAGE, electro-blotted, and detected by the indicated antibodies. (B) HEK293T cells were transfected with FLAG-tagged RIPK4 as indicated and treated for 4 hr with DMOG 24 hr post transfection. The cells were lysed, FLAG-RIPK4 immunoprecipitated, and proteins were separated by PAGE, electro-blotted, and detected by the indicated antibodies. (C) Graphs showing endogenous HSP90 interacting with exogenous FLAG-RIPK4 in the presence/absence of DMOG or overexpressed FIH. Bar graphs representing LFQ-intensity values normalized to the RIPK4 input are shown. The error bars represent SD and n = 6. (D) HEK293T cells were transfected with FIH siRNA or non-targeting siRNA and 24 hr later re-transfected with vector, TCF/LEF luciferase reporter TOPFLASH-8, β-Gal, FLAG-tagged RIPK4 or KD K51R mutant, and with or without V5-FIH or treated for 4 hr with DMOG 24 hr post transfection. The cells were lysed and the luciferase and β-Gal activity was measured. The bar graphs represent the luciferase activity normalized by β-Gal activity of three independent experiments with three biological replicates each (n = 9). The error bars are SD (p value < 0.05 = ^∗^ < 0.01 = ^∗∗^). (E) Western blot control of (D). In addition, cytoplasmatic β-catenin was enriched by removing glycosylated proteins with ConA beads. The supernatant was blotted and cytoplasmatic β-catenin was detected with an anti-β-catenin antibody. (F) Quantification of RIPK4 phosphorylation sites. The HEK293T cells were transfected with FLAG-tagged WT or KD RIPK4 and immunoprecipitated, digested, and analyzed by mass spectrometry. The phosphorylation sites were identified by searching against a human database and subsequently quantified by LFQ bar graphs representing LFQ-intensity values normalized to the unmodified peptides. The numbers on the x axis are the phosphorylation sites detected for the UniProt entry B7ZAU7. The error bars represent SEM and n = 6. (G) Quantification of RIPK4 phosphorylation sites. The HEK293T cells were transfected with FLAG-tagged WT RIPK4 with and without the FIH, and 24 hr after transfection, were treated for 4 hr with DMOG and immunoprecipitated, digested, and analyzed by mass spectrometry. The phosphorylation sites were identified by searching against a human database and subsequently quantified by LFQ bar graphs representing LFQ-intensity values normalized to the unmodified peptides. The numbers on the x axis are the phosphorylation sites detected for the UniProt entry B7ZAU7. The error bars represent SEM and n = 6.

In order to determine how hydroxylation of RIPK4 by FIH may affect the function of the substrates, we compared how the interactome changed in response to FIH overexpression and DMOG treatment. Both conditions are the extremes with respect to the hydroxylation status, and it is therefore plausible that changes in hydroxylation-dependent protein-protein interactions would be most significant between these two sets. Initially, we confirmed that we could reproduce that hydroxylase inhibition enhanced the interaction between RIPK4 and FIH when overexpressed (Figure 5A). Unfortunately, we were unable to confirm the interaction between endogenous FIH and endogenous RIPK4 as neither FIH nor RIPK4 antibody immunoprecipitated the bait protein with sufficient efficiency. Nonetheless, we were able to identify endogenous FIH in a FLAG-RIPK4 immunoprecipitation (IP) (Figure 5B) and endogenous RIPK4 in a V5-FIH IP (Figure S6D). We are therefore confident that the interaction is physiological.

Additionally to the induction of the FIH/RIPK4 complex, we noticed that chaperones such as HSP90 and members of the T-complex protein complex decreased their association with RIPK4 upon FIH overexpression (Figures 5C and S6A), as did three members of the SCF complex, BTRC, FBXW11, and SKP1 (Figure S6B). As we were only able to observe a significant regulation of the interaction when we overexpressed FIH, these changes within the RIPK4 complex may be due to altered hydroxylation levels or could be caused by FIH displacing proteins by tightly binding to the AR. To distinguish between either, we decided to test both hypotheses by either overexpressing FIH or inhibiting the hydroxylase activity in the follow-up experiments. The interaction with chaperones is an indication that the protein is in a flexible, thermodynamically less stable conformation. Because protein structure and flexibility can affect enzymatic activity (Taipale et al., 2013), we hypothesized that hydroxylation may affect RIPK4’s intrinsic kinase activity by regulating the stability of the C-terminal regulatory domain. The SCF complex on the other hand is involved in the degradation of signaling proteins such as β-catenin and NFκB (Chen, 2005, Winston et al., 1999). The FIH-dependent reduced interaction with the SCF complex suggested that RIPK4 protein stability might be regulated in a hydroxylation-dependent manner.

Neither overexpression of FIH nor incubation with DMOG influenced RIPK4 protein levels (Figure S6C). Thus, ruling a hydroxylation-dependent degradation out. To test if kinase activity of RIPK4 was regulated by hydroxylation, we relied on the fact that RIPK4 overexpression has been shown to activate β-catenin-dependent transcription, as well as inducing cytoplasmatic β-catenin levels (Huang et al., 2013). As both inductions are dependent on RIPK4 kinase activity, altered kinase activity should translate into enhanced or inhibited TCF/LEF transcriptional activity and cytoplasmatic β-catenin. We therefore co-transfected cells with TOPFLASH, a TCF/LEF luciferase reporter, vector kinase-dead RIPK4 (KD), and WT RIPK4 in conjunction with V5-FIH or FIH siRNA. As an additional control, we treated cells with 2 mM DMOG for 4 hr prior to lysis. Lysates were split, with one set analyzed for luciferase activity, one fraction analyzed for cytoplasmatic β-catenin (Huang et al., 2013), and a final fraction was lysed and used to determine expression levels.

As previously reported, activation of TCF/LEF transcriptional activity is induced by WT RIPK4, when compared to KD (Figures 5D and 5E). In addition, we observed that incubation with DMOG or FIH knock down reduced TCF/LEF-driven luciferase activity. Similarly, overexpression of V5-FIH was able to significantly increase luciferase activity (Figure 5D). As previously shown (Huang et al., 2013), WT RIPK4 induced non-membrane-bound, β-catenin levels. This induction was ablated by KD, DMOG, or FIH siRNA. To confirm that the observation that FIH regulates RIPK4-driven TCF/LEF transcriptional activity in other systems, we repeated the luciferase reporter assay in RKO cells, a colon cancer cell line which has not been shown to have a mutated Wnt-signaling pathway. As expected, expression of RIPK4 increased TCF/LEF-driven luciferase expression when compared to a vector control. The induction was completely ablated when we knocked down FIH by siRNA (Figure S7A). Taken together, these data demonstrated that FIH-dependent hydroxylation stimulates RIPK4 signaling in RKO and HEK293T cells. RIPK4 has also been shown to be autophosphorylated (Meylan et al., 2002), we therefore decided to quantify RIPK4 kinase activity by quantifying kinase activity-dependent RIPK4 phosphorylation sites. Initially, we compared the phosphorylation status of WT and KD RIPK4. We identified several phosphorylation sites of which some were absent in KD mutant (Figure 5F), indicating that the phosphorylation of these sites required RIPK4 kinase activity. Next, we quantified the phosphorylation status of WT RIPK4 in the presence or absence of DMOG and when V5-FIH was overexpressed (Figure 5G). We quantified the phosphorylation sites by LFQ and were able to detect that DMOG inhibition and FIH overexpression altered the phosphorylation on sites which were determined to be kinase dependent in the previous assay. Overall, these data demonstrated that hydroxylation and FIH regulate RIPK4 kinase-dependent phosphorylations.

Taken together, we demonstrated that FIH binds to RIPK4 and that hydroxylase inhibition and FIH-driven hydroxylation affects RIPK4 activity and downstream signaling.

### Biological Consequence of PHD3-Dependent Hydroxylation of MAPK6

**Figure 6:**
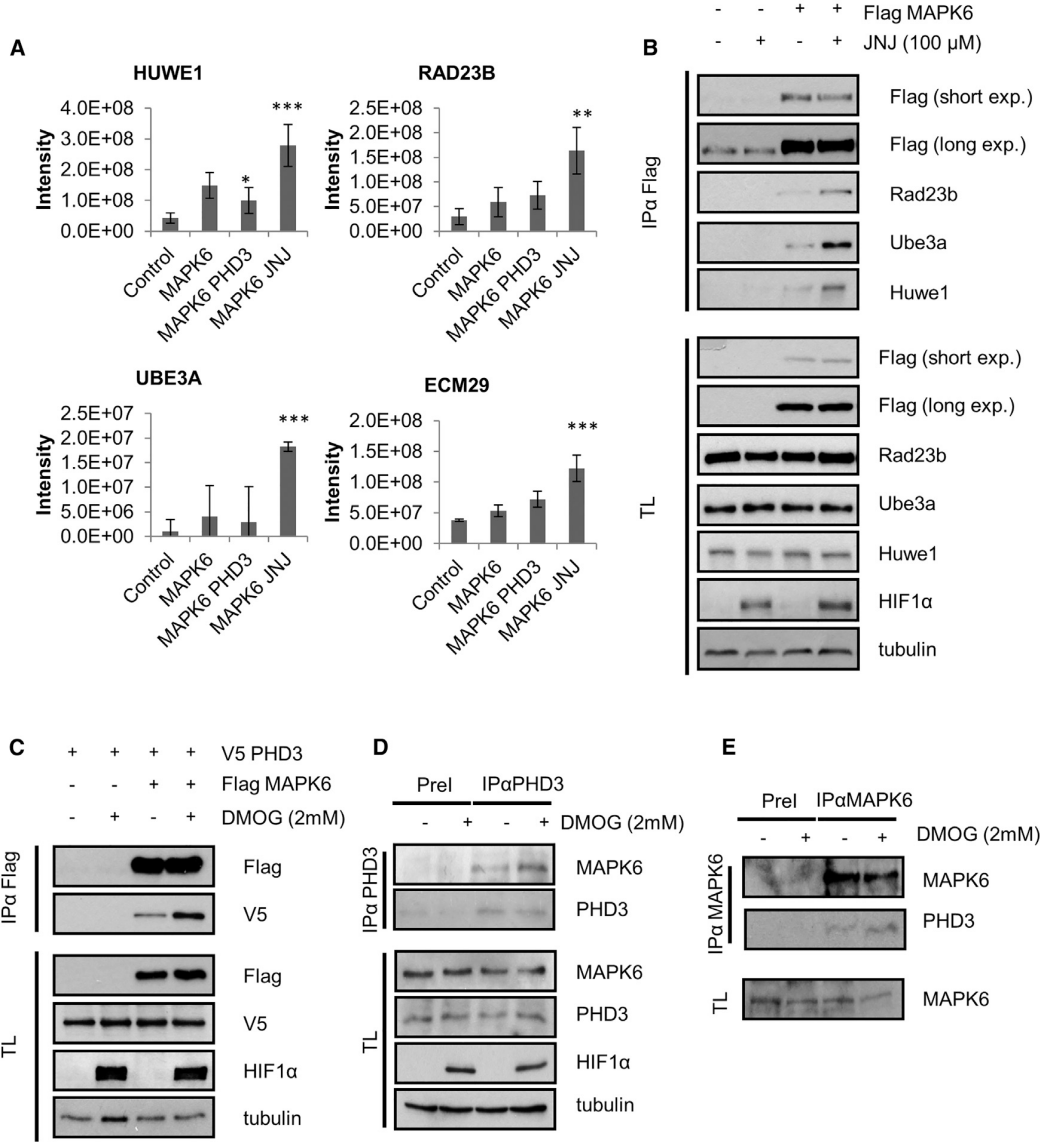
MAPK6 Interaction Screen (A) Graphs showing selected MAPK6 interactions which specifically change upon treatment with JNJ for 4 hr. The bar graphs representing LFQ-intensity values normalized to the MAPK6 input are shown. The error bars represent SD and n = 6. (B) HEK293T cells were transfected with FLAG-MAPK6 and treated 24 hr post-transfection for 3 hr with JNJ. The cells were lysed, FLAG-MAPK6 immunoprecipitated, and proteins were separated by PAGE, electro-blotted, and detected by the indicated antibodies. (C) HEK293T cells were transfected with FLAG-tagged MAPK4 and V5-PHD3. At 24 hr post transfection, cells were treated for 2 hr with DMOG. The cells were lysed, FLAG-MAPK6 immunoprecipitated, and proteins were separated by PAGE, electro-blotted, and detected by the indicated antibodies. (D) HEK293T cells were treated for 2 hr with DMOG. The cells were lysed, PHD3 immunoprecipitated, and proteins were separated by PAGE, electro-blotted, and detected by the indicated antibodies. (E) HEK293T cells were treated for 2 hr with DMOG. The cells were lysed, MAPK6 immunoprecipitated, and proteins were separated by PAGE, electro-blotted, and detected by the indicated antibodies.

Subsequently, we analyzed the MAPK6 interaction data set and 15 proteins were significantly affected by hydroxylase inhibition. Of these, four have been linked to ubiquitination (HUWE1 and UBE3A), ubiquitin recognition (RAD23b), and the proteasome (ECM29) (Figures 6A and 6B). Considering these data, it was a reasonable hypothesis reduced hydroxylation leads to an ubiquitin directed proteasomal degradation of MAPK6 by HUWE1 and UBE3A.

**Figure 7:**
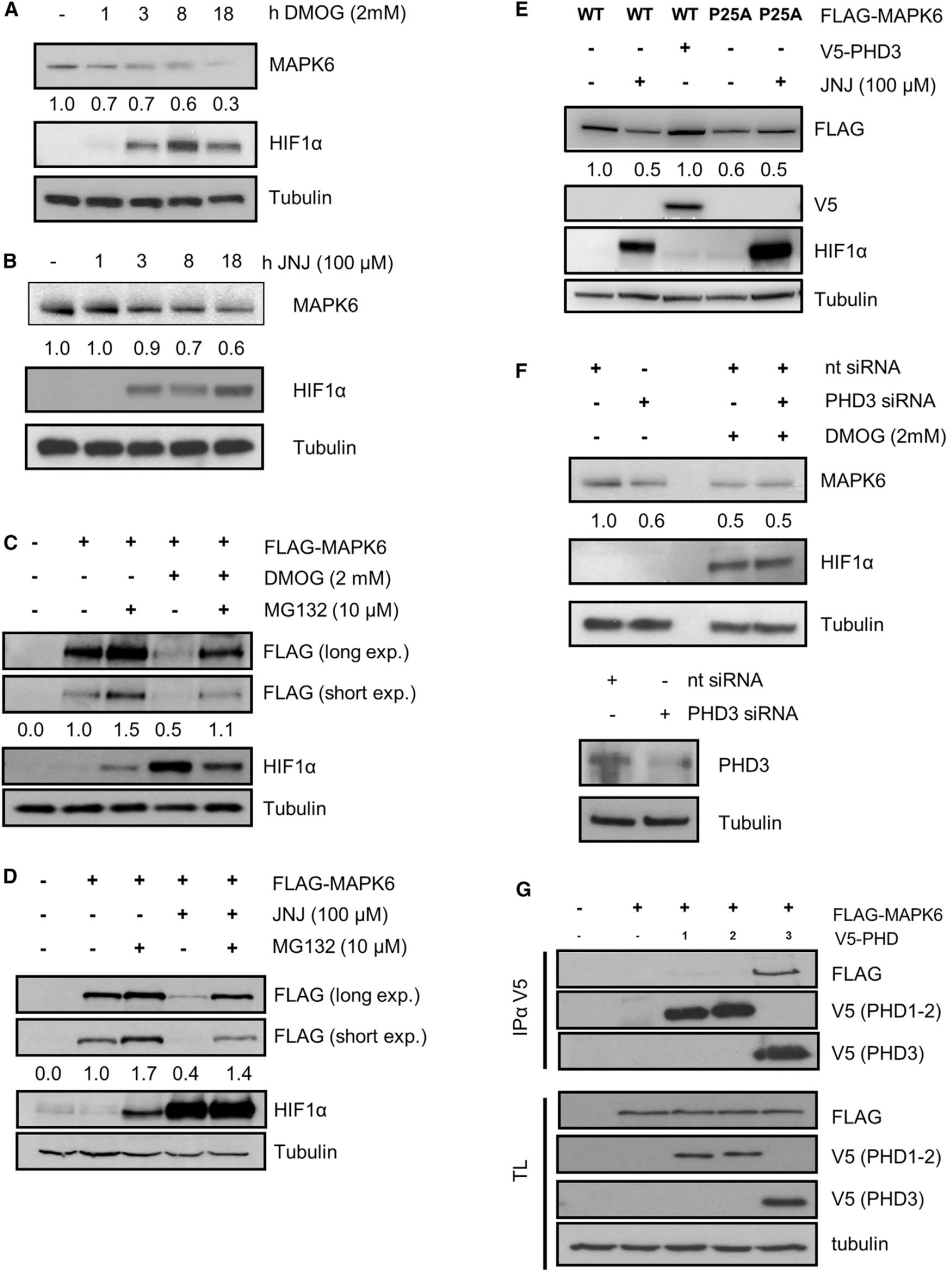
PHD3 Stabilizes MAPK6 by Hydroxylating Pro25 (A) HEK293T cells were treated for indicated times with DMOG. The cells were lysed and proteins were separated by PAGE, electro-blotted, and detected by the indicated antibodies. The western blot bands corresponding to MAPK6 were quantified and normalized against tubulin levels. (B) HEK293T cells were treated for indicated times with JNJ. The cells were lysed and proteins were separated by PAGE, electro-blotted, and detected by the indicated antibodies. The western blot bands corresponding to MAPK6 were quantified and normalized against tubulin levels. (C) HEK293T cells were transfected with FLAG-tagged MAPK4 and 24 hr post transfection, cells were treated for 8 hr with DMOG and/or MG132. The cells were lysed, proteins were separated by PAGE, electro-blotted, and detected by the indicated antibodies. The western blot bands corresponding to FLAG-MAPK6 were quantified and normalized against tubulin levels. (D) HEK293T cells were transfected with FLAG-tagged MAPK4 and 24 hr post transfection, cells were treated for 8 hr with JNJ and/or MG132. The cells were lysed, proteins were separated by PAGE, electro-blotted, and detected by the indicated antibodies. The western blot bands corresponding to FLAG-MAPK6 were quantified and normalized against tubulin levels. (E) HEK293T cells were transfected with FLAG-tagged MAPK6 or the P25A mutant with and without V5-PHD3 and treated for 6 hr with JNJ 24 hr post transfection. The cells were lysed and proteins were separated by PAGE, electro-blotted, and detected by the indicated antibodies. The western blot bands corresponding to FLAG-MAPK6 were quantified and normalized against tubulin levels. (F) HEK293T cells were transfected with PHD3 siRNA or non-targeting siRNA and treated for 6 hr with DMOG 48 hr post transfection. The cells were lysed and proteins were separated by PAGE, electro-blotted, and detected by the indicated antibodies. The western blot bands corresponding to MAPK6 were quantified and normalized against tubulin levels. (G) HEK293T cells were transfected with FLAG-tagged MAPK6 with and without V5-PHD1, 2, or 3 as indicated. The cells were lysed, V5-tagged proteins were immunoprecipitated and separated by PAGE, electro-blotted, and detected by the indicated antibodies.

Initially, we confirmed that PHD3 interacted with MAPK6 at the exogenous as well as endogenous level and that the interaction was inducible by DMOG (Figures 6C6E). Second, to test whether hydroxylase inhibition increases proteasomal degradation of MAPK6, we treated cells with two structurally unrelated hydroxylase inhibitors (DMOG and JNJ) and quantified the expression levels of endogenous MAPK6 by western blotting (Figures 7A and 7B). In line with our hypothesis, MAPK6 protein levels decreased in a linear fashion over the duration of the treatment with either inhibitor. To ascertain that the decrease of the MAPK6 was due to proteasomal degradation, we transfected cells with FLAG-MAPK6 and treated the cells with DMOG or JNJ in the presence or absence of the proteasomal inhibitor MG132 (Figures 7C and 7D). Incubation with MG132 was able to stabilize and hydroxylase inhibition to reduce MAPK6 protein levels. When cells were treated with DMOG or JNJ and MG132 simultaneously, no decrease in MAPK6 level was observable.

To confirm that the hydroxylase inhibitor-dependent degradation was mediated by hydroxylation on Pro25, we mutated the site to an alanine (P25A) and transfected WT and P25A MAPK6 into HEK293T cells and treated the cells for 8 hr with JNJ or DMOG (Figures 7E and S7B). Incubation with JNJ or DMOG reduced exogenous levels of MAPK6, whereas overexpression of V5-PHD3 did not increase MAPK6 protein levels. On the other hand, protein levels of the P25A mutant expressed at lower levels, when compared to the WT, and, crucially, were not further suppressed by 8 hr of either inhibitor. Intriguingly, expression of mutant and WT MAPK6 could be increased to equal levels by blocking proteasomal degradation, suggesting that the differential expression levels are due to enhanced degradation of the mutant (Figure S7B).

Having established that Pro25 regulates the expression levels of MAPK6 in a hydroxylase-dependent manner, we wanted to ensure that PHD3 is an essential regulator of MAPK6 under endogenous, normoxic conditions. Therefore, we reduced cellular PHD3 levels by siRNA. Reassuringly, we observed a robust reduction of endogenous MAPK6. Moreover, treatment with the pan-hydroxylase inhibitor DMOG was unable to further suppress the expression of MAPK6 (Figure 7F). Next, we determined if any of the other PHDs were able to interact with MAPK6. We expressed FLAG-MAPK6 in the presence of a vector control or V5-tagged PHD1, 2, or 3. We immunoprecipitated the hydroxylase and were only able to detect the interaction between MAPK6 and PHD3 (Figure 7G). Taken together, both these data demonstrated that PHD3 is the main MAPK6-hydroxylase in HEK293T cells under normoxic conditions.

In conclusion, we confirmed that MAPK6 interacts specifically with PHD3, that PHD3-dependent hydroxylation of Pro25 of MAPK6 regulates its protein stability, and that PHD3 is the endogenous enzyme which regulates MAPK6 protein levels.

## Discussion

Our data suggest that these oxygen-dependent enzymes regulate multiple signaling pathways by means of a distributed control, which is in contrast to the paradigm that hydroxylases regulate predominantly the HIF-pathway by exercising their control on the master switch. Interestingly, a substantial set of proteins in the canonical Wnt-pathway were identified as potential substrates. Given that these proteins are members of multiprotein complexes, it is likely that only some of them are directly interacting with, and are substrates of, PHD3. Nevertheless, this is a clear indication that β-catenin signaling is regulated by both FIH and PHDs. This is intriguing, as comparative oxygen and β-catenin gradients have been reported in colonic crypts, where low oxygen correlates with low nuclear β-catenin. Our initial data on RIPK4 induced TCF/LEF transcriptional activity appears to support this connection. We also confirmed that MAPK6 is hydroxylated by PHD3 close to two N-terminal domains which regulate protein degradation (Ulyatt et al., 2011) In contrast to many PHD substrates, hydroxylation of MAPK6 on Pro(25) stabilizes the protein. MAPK6 has recently been reported to control the expression of VEGFR2 (Wang et al., 2014), and it is therefore possible that the suppression of the protein by hypoxia may switch in the expression the VEGFR isoforms, which has been indeed observed in low oxygen (Ulyatt et al., 2011). The question still arises as to how MAPK6 may regulate VEGFR2 expression. Based on our interaction data, MAPK6 interacts specifically with IRAK1, a protein involved in interleukin signaling, and the MAPK cascade proteins PRAK, Raf-1, and BRAF, suggesting an involvement in MAPKs and NFκB signaling pathways. As VEGFR2 mRNA expression is regulated by NFκB (González-Pacheco et al., 2006), it is plausible that this pathway provides the link to MAPK6.

Over the past years, several attempts have been made to systematically screen hydroxylation sites of endogenous proteins. Although these approaches had some success, identifying hydroxylation sites remains a formidable task. This is in stark contrast to identification of phosphorylations, acetylations, and ubiquitination sites which benefit from availability of affinity based enrichment methods at the peptide and protein level (Olsen and Mann, 2013). Further complicating the analysis, hydroxylations and oxidations can occur on a multitude of amino acid side chains. Thus, in order to assign the site correctly within a peptide, fragmentation data have to be of high resolution and coverage to give confidence in the assignment of the site. We have overcome this issue by using high resolution and mass accuracy HCD spectra as well as adding oxidations of nine individual amino acid side chains as variable modification. The inclusion of this array of oxidations permits to determine localization probabilities in an unbiased manner and reduces the need for visual inspection of the fragmentation spectra. In the absence of an efficient systematic screening and enrichment method for hydroxylations, we conclude that a targeted screen is currently the best way to identify regulated hydroxylations and to determine the function in an unbiased manner.

Our results indicate that a cellular assay followed by quantitative mass spectrometry is a viable way to determine enzymatic regulation of hydroxylation sites. We also attempted to confirm the hydroxylase specificities in vitro with purified substrates. The outcomes of experiments with biotinylated peptides were disappointing. We easily detected oxidized/hydroxylated peptides, but exclusively oxidized on the biotin residue. Fortunately, as we analyzed the assay by liquid chromatography (LC)-MS/MS, we were able to detect this false positive result, and based on these data, we would not recommend performing this assay without such a setup, as analyzing the assay by MALDI-MS would not reveal these issues. Because we were unable to hydroxylate peptides in vitro, we switched to IVT-full-length proteins and obtained much more encouraging results. We were able to confirm that MAPK6 and RIPK4 can be hydroxylated in vitro. Disappointingly, we failed to confirm all four FIH-mediated hydroxylations in RIPK4, despite strong evidence in cells. Based on our observations, we conclude that in vitro assays have to be tailored to the individual substrate in order to avoid potential false positives and negatives.

## Experimental Procedures

### Cell Culture

HEK293T cells were cultured in Dulbecco’s modified Eagle medium (DMEM) supplemented with 2 mM glutamine (Invitrogen) and 10% fetal calf serum (Invitrogen). Plasmids and siRNA oligonucleotides were transfected with Lipofectamine 2000 (Invitrogen) according to the vendor’s instructions.

### Immunoblotting

Total lysates and affinity precipitates were fractionated by SDS-PAGE and transferred onto nitrocellulose filters. Immunocomplexes were visualized by enhanced chemiluminescence detection (GE Healthcare) with horseradish peroxidase-conjugated secondary antibodies (Bio-Rad Laboratories). Experiments were repeated at least three times.

### Mass Spectrometry and Immunoprecipitations

Samples were generated and processed as described. For interaction data: Turriziani et al. (2014) and for expression data: Farrell et al. (2014). Variable modifications were N-terminal acetylation (protein) and oxidation (M) for the interaction and expression screen and oxidation (MWYFKPHDN) for the hydroxylation screen.

### Bioinformatic Analysis

Uniprot accession numbers were reduced to one entry per protein group and uploaded either into the IPA (http://www.ingenuity.com/), StringDB (http://www.string-db.org/), or DAVID Bioinformatics Resources (https://www.david.ncifcrf.gov/). IPA was used to identify enriched pathways, and DAVID to identify enriched protein domains. String output was limited to “experimental data” and stringency was set to “moderate”.

## Author Contributions

Conceived and designed the experiments: A.v.K. and C.T.T.; performed the experiments: J.R., R.P., A.G.M., N.R., and A.H.; mass spectrometric analysis: J.R. and S.K.; analyzed the data: J.R. and A.v.K.; mathematical model: L.K.N.; and wrote the paper: A.v.K., J.R., L.K.N., and N.M..

## Supplemental Information

Document S1. Supplemental Experimental Procedures and Figures S1–S7

Table S1. FIH Immunoprecipitation Data, Related to Figure 2 The table shows HEK293T cells that were transfected with V5-FIH or an empty vector and 48 hr post-transfection treated or not for 4 hr with DMOG. The cells were lysed and V5-FIH was immunoprecipitated and digested on-beads. The peptides were identified by mass spectrometry and quantified by LFQ by the MaxQuant software package. The contaminants and reverse data base hits were deleted. (sheet 1, raw data) The LFQ data, as determined by the MaxQuant software. The column AD is the t test of the untreated FIH (columns M–R) versus the negative control (columns A–F). The column AE is the t test of the DMOG-treated FIH (columns S–X) versus the negative control (columns G–L). The column AG is the ratio of the averages of the untreated FIH/negative control intensities. The column AH is the ratio of the averages of the DMOG-treated FIH/negative control intensities. Highlighted in “orange” are proteins which are statistically enriched in the FIH samples under either condition (p < 0.01 and ratio >2). (sheet 2, FIH interactome) The subset of proteins statistically enriched in the FIH DMOG-treated sample versus the negative control from sheet 1 (p <0.01 and ratio >2). The LFQ intensities are normalized to the FIH-input. Highlighted in “orange” are proteins which are statistically enriched in the FIH DMOG sample versus the FIH untreated sample. The protein expression changes as determined by whole cell LFQ expression proteomics. The experiments were run as biological triplicate, NaN indicates protein was not identified (X–AE). (sheet 3, FIH DMOG induced) The normalized LFQ-intensities of potential FIH substrates are shown. The columns X –AE are the averages and SD of the intensities and are used for the bar graph in Figures 2 and S2.

Table S2. PHD3 Immunoprecipitation Data, Related to Figure 2 The table shows HEK293T cells that were transfected with V5-PHD3 or an empty vector and 48 hr post-transfection treated or not for 4 hr with DMOG. The cells were lysed and V5-PHD3 was immunoprecipitated and digested on-beads. The peptides were identified by mass spectrometry and quantified by LFQ by the MaxQuant software package. The contaminants and reverse data base hits were deleted. (sheet 1, raw data) The LFQ data as determined by MaxQuant. The column AD is the t test of the untreated PHD3 (columns M–R) versus the negative control (columns A–F). The column AE is the t test of the DMOG-treated PHD3 (columns S–X) versus the negative control (columns G–L). The column AG is the ratio of the averages of the untreated PHD3/negative control intensities. The column AH is the ratio of the averages of the DMOG-treated PHD3/negative control intensities. Highlighted in “orange” are proteins which are statistically enriched in the PHD3 samples under either condition (p < 0.01 and ratio >2). (sheet 2, PHD3 interactome) The subset of proteins statistically enriched in the PHD3 DMOG-treated sample versus the negative control from sheet 1 (p < 0.01 and ratio >2). The LFQ intensities are normalized to the PHD3-input. Highlighted in “orange” are proteins which are statistically enriched in the PHD3 DMOG sample versus the PHD3 untreated sample (column AF p < 0.01 and column AI ratio >1.5). The protein expression changes as determined by whole cell LFQ expression proteomics are shown. The experiments were run as biological triplicate, NaN indicates protein was not identified (X–AE). (sheet 3, PHD3 DMOG induced) The normalized LFQ-intensities of potential PHD3 substrates are shown. Columns X– AE are the averages and SD of the intensities and are used for the bar graph in Figures 2 and S2.

Table S3. RIPK4 Interactome, Related to Figure 5 The table shows HEK293T cells that were transfected with FLAG-RIPK4 or empty vector and an empty vector or V5-FIH and 48 hr post-transfection treated or not for 4 hr with DMOG. The cells were lysed and FLAG-RIPK4 was immunoprecipitated and digested on-beads. The peptides were identified by mass spectrometry and quantified by LFQ by the MaxQuant software package and normalized to the RIPK4 input. The columns A–X are the LFQ intensities as determined by MaxQuant. The columns A–F are the untreated negative control empty vector, columns G–L FLAG-RIPK4 and FIH overexpressing, and columns M–R untreated FLAG-RIPK4 overexpressing and S–X are DMOG-treated FLAG-RIPK4 overexpressing samples. The columns Y–AA show the p value of the t test of the FLAG-RIPK4 overexpressing samples versus the negative control and columns AB–AD show the ratios of the average LFQ intensities of the FLAG-RIPK4 overexpressing samples over the negative control. The columns AE and AG show the p value of the t test of the FLAG-RIPK4 overexpressing untreated or FIH overexpressing versus the negative DMOG-treated sample. The columns AF and AH are the corresponding average ratios.

Table S4. MAPK6 Interactome, Related to Figure 6 The table shows HEK293T cells that were transfected with FLAG-MAPK6 or empty vector and an empty vector or HA-PHD3 and 48 hr post-transfection treated or not for 4 hr with DMOG. The cells were lysed and FLAG-MAPK6 was immunoprecipitated and digested on-beads. The peptides were identified by mass spectrometry and quantified by LFQ by the MaxQuant software package and normalized to the MAPK6 input. The columns A–X are the LFQ intensities as determined by MaxQuant. The columns A–F are the untreated negative control empty vector, the columns G–L are the untreated FLAG-MAPK6 overexpressing, the columns M–R are JNJ treated FLAG-MAPK6 overexpressing, and the columns S–X FLAG-MAPK6 and HA-PHD3 are overexpressing samples. The columns Y–AA show the p value of the t test of the FLAG-MAPK6 overexpressing samples versus the negative control and columns AB–AD show the ratios of the average LFQ intensities of the FLAG-MAPK6 overexpressing samples over the negative control. The columns AE and AF show the p value of the t test of the FLAG-MAPK6 overexpressing untreated or HA-PHD3 overexpressing versus the negative JNJ treated sample. The columns AG and AH are the corresponding average ratios.

Document S2. Article plus Supplemental Information
