# Supplementary material for: Substrate-Trapped Interactors of PHD3 and FIH Cluster in Distinct Signaling Pathways
Source: Cell Rep. 2016 Mar 10;14(11):2745–60. doi: 10.1016/j.celrep.2016.02.043 (PMC4805855; doi:10.1016/j.celrep.2016.02.043)
Supplement: Document S1. Supplemental Experimental Procedures and Figures S1–S7 [file mmc1.pdf]

Cell Reports, Volume *14*

Supplemental Information

Substrate-Trapped Interactors of PHD3 and FIH

Cluster in Distinct Signaling Pathways

Javier Rodriguez, Ruth Pilkington, Amaya Garcia Munoz, Lan K. Nguyen, Nora Rauch, Susan Kennedy, Naser Monsefi, Ana Herrero, Cormac T. Taylor, and Alex von Kriegsheim

Figure S1

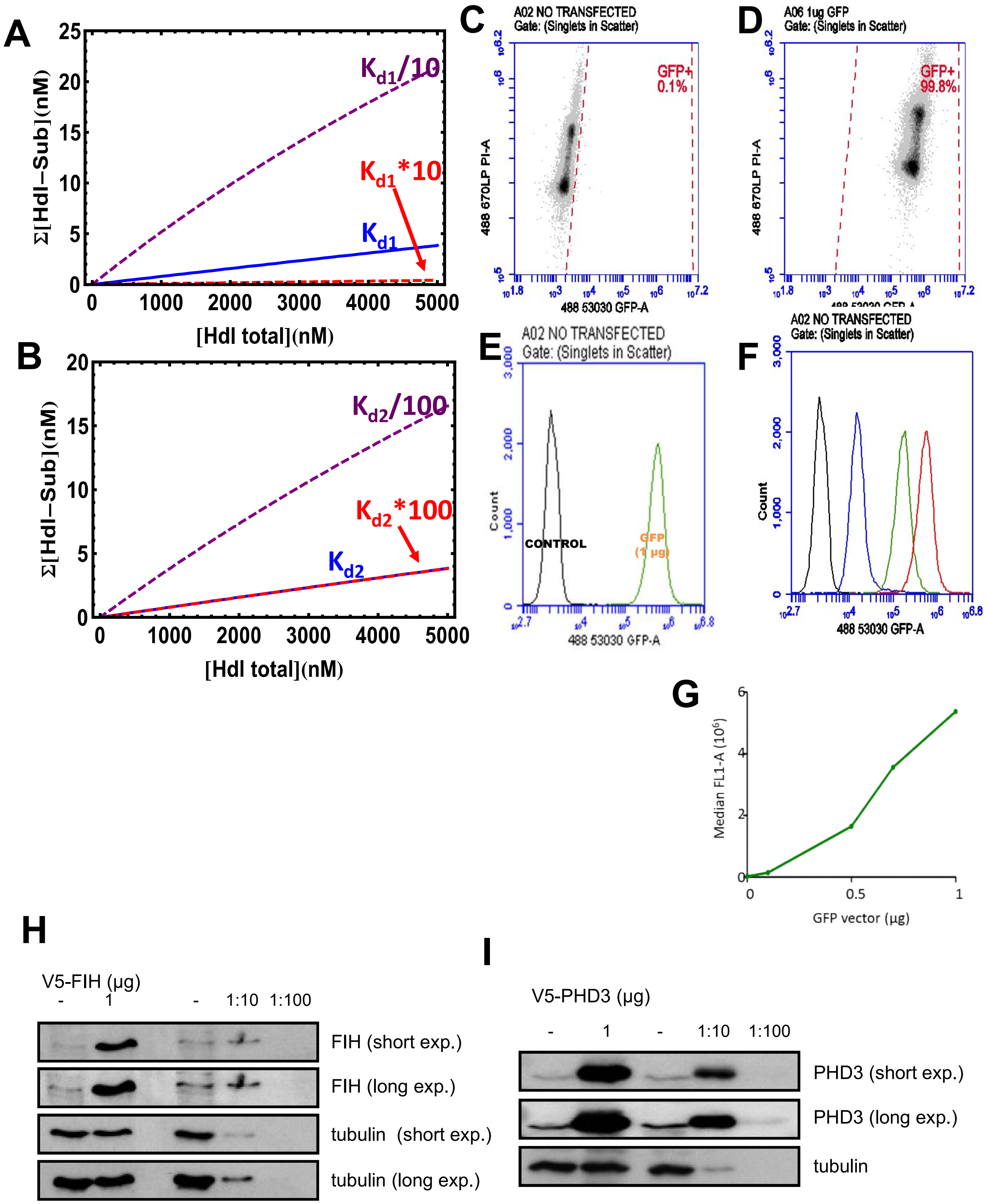

A
Σ[Hdl−Sub](nM)
[Hdl total](nM)
Kd1/10
Kd1*10
Kd1
B
Σ[Hdl−Sub](nM)
[Hdl total](nM)
Kd2/100
Kd2*100
Kd2
C
A02 NO TRANSFECTED
Gate: (Singlets in Scatter)
GFP+
0.1%
488 670LP PI-A
488 53030 GFP-A
D
A06 1ug GFP
Gate: (Singlets in Scatter)
GFP+
99.8%
488 670LP PI-A
E
A02 NO TRANSFECTED
Gate: (Singlets in Scatter)
CONTROL
GFP
(1 µg)
Count
488 53030 GFP-A
F
A02 NO TRANSFECTED
Gate: (Singlets in Scatter)
Count
488 53030 GFP-A
G
Median FL1-A (10^6)
GFP vector (µg)
H
V5-FIH (µg)
- 1 - 1:10 1:100
FIH (short exp.)
FIH (long exp.)
tubulin (short exp.)
tubulin (long exp.)
I
V5-PHD3 (µg)
- 1 - 1:10 1:100
PHD3 (short exp.)
PHD3 (long exp.)
tubulin

Figure S2

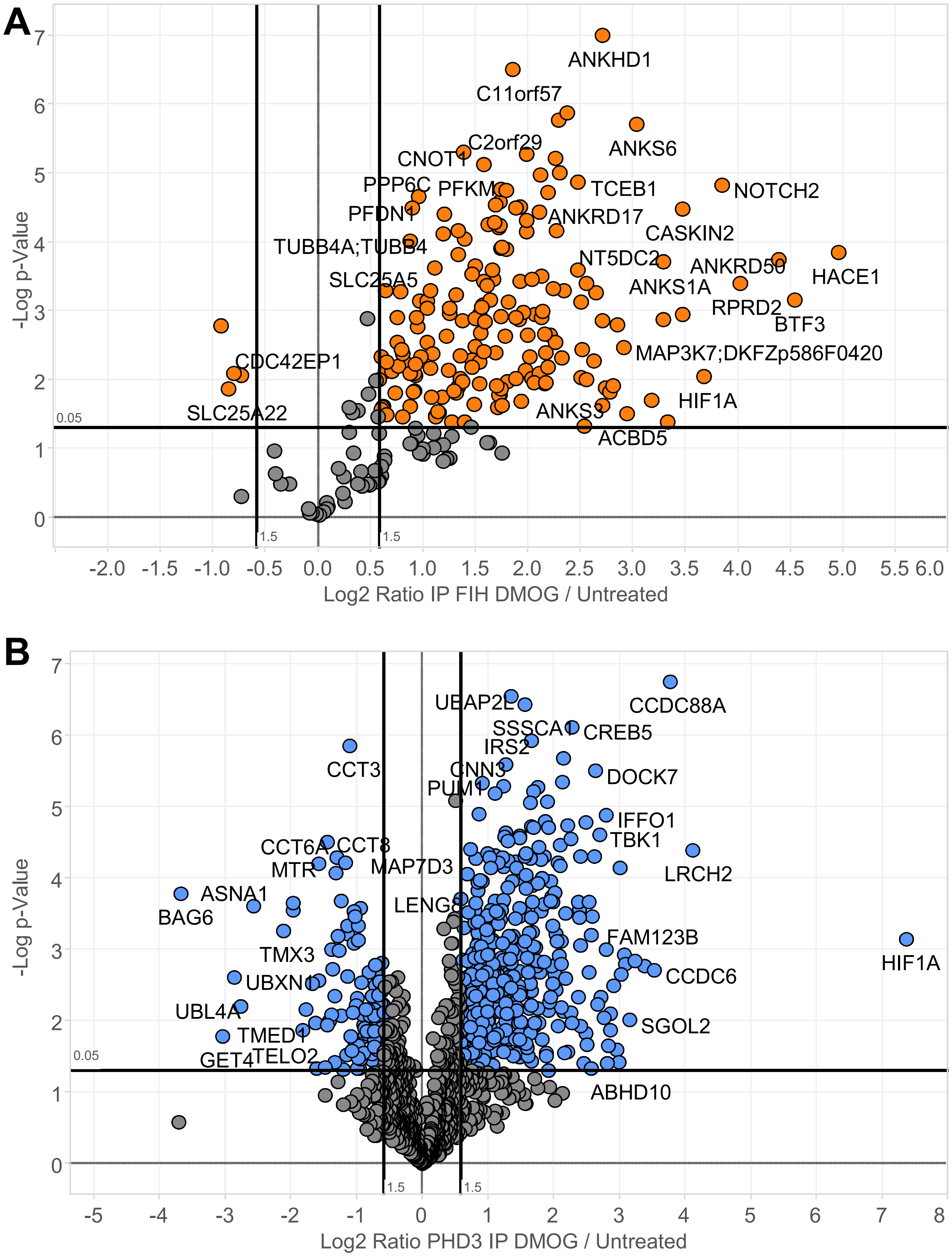
A
ANKHD1
C11orf57
C2orf29
ANKS6
CNOT1
PPP6C
PFKM
TCEB1
NOTCH2
PFDN1
ANKRD17
CASKIN2
TUBB4A;TUBB4
NT5DC2
ANKRD50
HACE1
SLC25A5
ANKS1A
RPRD2
BTF3
MAP3K7;DKFZp586F0420
CDC42EP1
HIF1A
SLC25A22
ANKS3
ACBD5
0.05
1.5
1.5
-Log p-Value
Log2 Ratio IP FIH DMOG / Untreated
B
UBAP2L
CCDC88A
SSSCA1
CREB5
IRS2
CCT3
CNN3
DOCK7
PUM1
IFFO1
TBK1
CCT6A
CCT8
LRCH2
MTR
MAP7D3
ASNA1
LENG8
BAG6
FAM123B
TMX3
HIF1A
CCDC6
UBXN1
UBL4A
SGOL2
TMED1
GET4
TELO2
ABHD10
0.05
1.5
1.5
-Log p-Value
Log2 Ratio PHD3 IP DMOG / Untreated

Figure S3

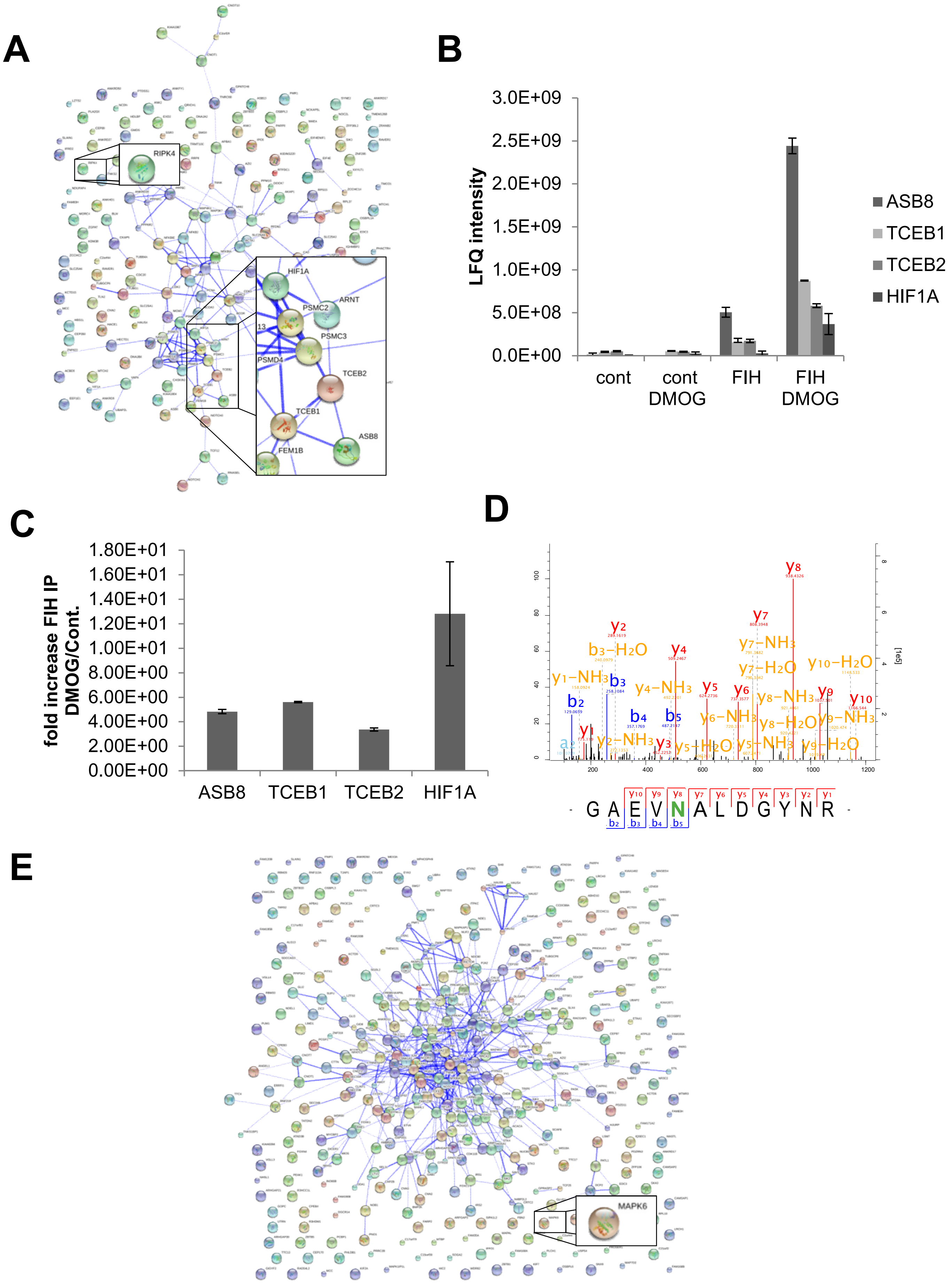
A
RIPK4
HIF1A
ARNT
PSMC2
PSMC3
PSMD4
TCEB2
TCEB1
ASB8
FEM1B
B
LFQ intensity
3.0E+09
2.5E+09
2.0E+09
1.5E+09
1.0E+09
5.0E+08
0.0E+00
cont
cont DMOG
FIH
FIH DMOG
ASB8
TCEB1
TCEB2
HIF1A
C
fold increase FIH IP DMOG/Cont.
1.80E+01
1.60E+01
1.40E+01
1.20E+01
1.00E+01
8.00E+00
6.00E+00
4.00E+00
2.00E+00
0.00E+00
ASB8
TCEB1
TCEB2
HIF1A
D
G A E V N A L D G Y N R
E
MAPK6

Figure S4

A

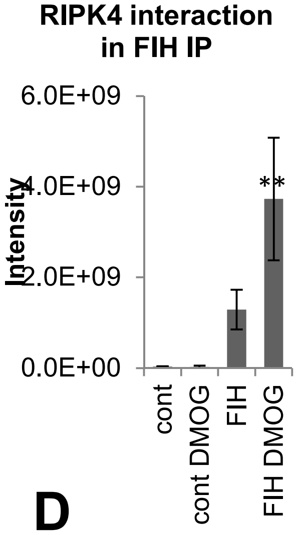

B

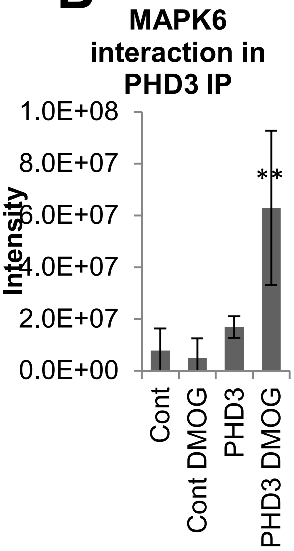

C

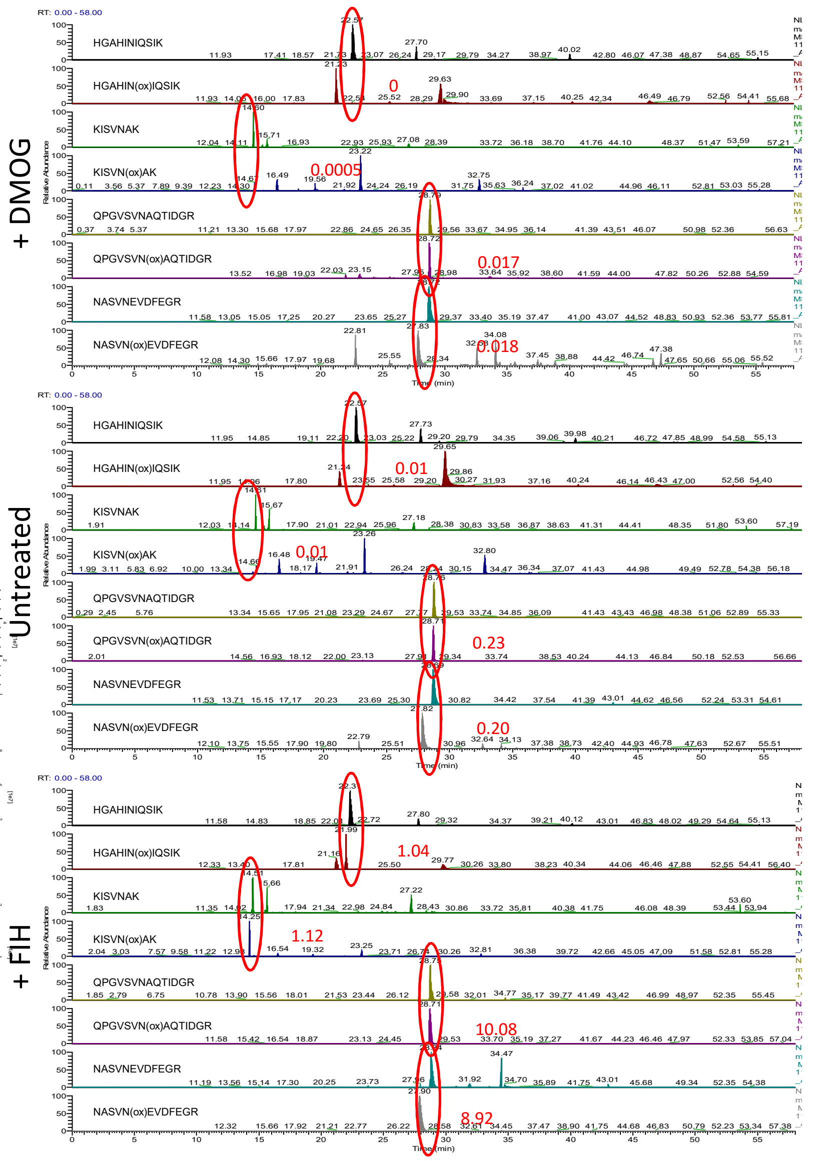

D

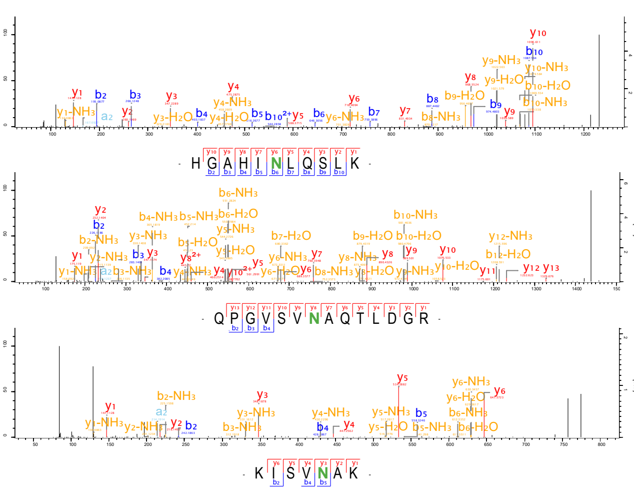

E

a ion		b²⁺ ion		b ion					y ion		y²⁺ ion	
Δ ppm	mass	Δ ppm	mass	Δ ppm	mass		seq		mass	Δ ppm	mass	Δ ppm
	110.1		138.1		138.1	1	H	11				
-2.2	167.1		195.1	+1.149	195.1	2	G	10	1097	-2.08	548.8	+7.15
	238.1		266.1	-2.01	266.1	3	A	9	1040	+0.091	520.3	+9.057
	375.2		403.2	-0.85	403.2	4	H	8	968.6	-1.18	484.8	+6.18
	488.3		516.3	-1.56	516.3	5	I	7	831.5	+0.826	831.5	
	618.3	-6.71	323.7	-2.85	646.3	6	N	6	718.4	-0.88	718.4	
	731.4	-4.49	380.2	-2.34	759.4	7	L	5	588.4	+0.388	588.4	
	859.5	+9.776	444.2	-2.85	887.4	8	Q	4	475.3	-1.36	475.3	
	946.5	-0.48	487.7	-3.46	974.5	9	S	3	347.2	-2.33	347.2	
	1060	+0.276	544.3	+0.816	1088	10	L	2	260.2	-1.97	260.2	
						11	K	1	147.1	-2.05	147.1	

b ion					y ion		y²⁺ ion	
Δ ppm	mass		seq		mass	Δ ppm	mass	Δ ppm
	115.05	1	N	12				
-1.6658	186.087	2	A	11	1238.56		1238.56	
-2.9755	273.119	3	S	10	1167.53	-2.6448	1167.53	
-2.4378	372.188	4	V	9	1080.5	-1.1049	1080.5	
	502.226	5	N	8	981.427	-2.3397	491.217	+2.7536
-4.1432	631.268	6	E	7	851.389	-4.2596	851.389	
	730.337	7	V	6	722.347	-1.0316	722.347	
	845.364	8	D	5	623.278	-2.6423	623.278	
	992.432	9	F	4	508.251	-1.3411	508.251	
	1121.47	10	E	3	361.183	-2.6479	361.183	
	1178.5	11	G	2	232.14	-2.2361	232.14	
		12	R	1	175.119	-0.9092	175.119	

a ion		b ion					y ion		y²⁺ ion	
Δ ppm	mass	Δ ppm	mass		seq		mass	Δ ppm	mass	Δ ppm
	101.0709		129.0659	1	Q	14				
-1.12953	198.1237	-2.31296	226.1186	2	P	13	1329.676	-5.31425	665.3415	+1.74367
	255.1452	+2.159024	283.1401	3	G	12	1232.623	-3.44957	1232.623	
	354.2136	-4.33334	382.2085	4	V	11	1175.601		1175.601	
	441.2456	+0.605197	469.2405	5	S	10	1076.533	-3.45202	1076.533	
	540.314		568.3089	6	V	9	989.501	-1.35221	989.501	
	670.3519	+4.352163	698.3468	7	N	8	890.4326	-2.61749	890.4326	
	741.389		769.3839	8	A	7	760.3948	-3.49087	760.3948	
	869.4476		897.4425	9	Q	6	689.3577	-0.86228	689.3577	
	970.4952		998.4901	10	T	5	561.2991	-0.55895	561.2991	
	1083.579		1111.574	11	L	4	460.2514	-1.83867	460.2514	
	1198.606		1226.601	12	D	3	347.1674	-2.4909	347.1674	
	1255.628		1283.623	13	G	2	232.1404	-2.48056	232.1404	
				14	R	1	175.119	+0.571286	175.119	

a ion		b²⁺ ion		b ion					y ion		y²⁺ ion	
Δ ppm	mass	Δ ppm	mass	Δ ppm	mass		seq		mass	Δ ppm	mass	Δ ppm
	101		129		129	1	K	7				
+0.27	214		242	+0.16	242	2	I	6	647	-1.4	324	+2.97
	301		329	+8.11	329	3	S	5	534	+1.54	534	
	400		428	+0.52	428	4	V	4	447	-3.2	447	
	530		558	+1.46	558	5	N	3	348	-2.2	348	
	601	-14	315		629	6	A	2	218	-2.7	218	
						7	K	1	147	-2.7	147	

Figure S5

A

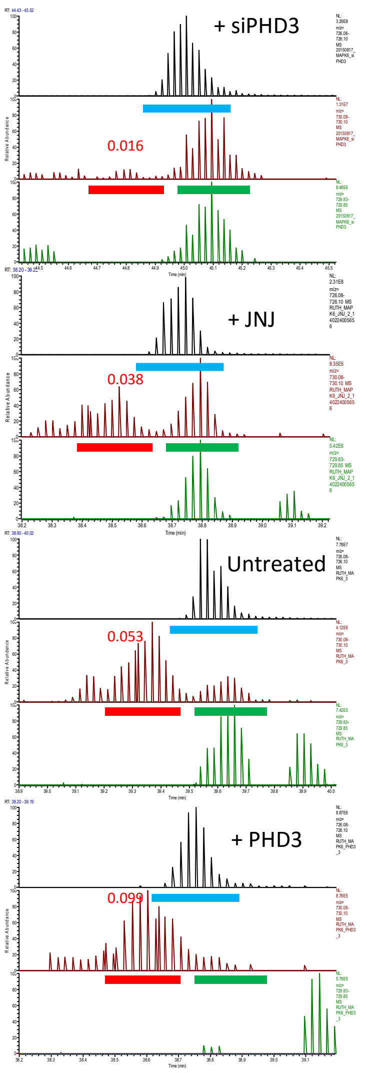

B

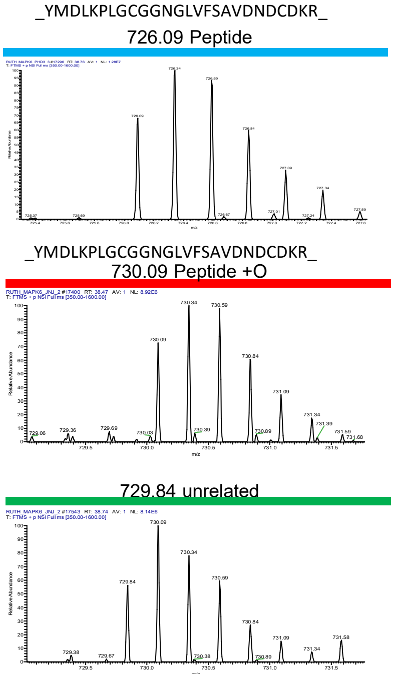

C

a^{2+} ion		a ion		b^{2+} ion		b ion					y ion		y^{2+} ion	
Δ ppm	mass	Δ ppm	mass	Δ ppm	mass	Δ ppm	mass		seq		mass	Δ ppm	mass	Δ ppm
	136.076	+0.47439	136.076		164.071		164.071	1	Y	26				
	267.116	-1.5009	267.116		295.111	+0.49726	295.111	2	M	25	2754.26		2754.26	
	382.143		382.143		410.138	+0.71441	410.138	3	D	24	2623.22	-0.8647	2623.22	
	495.227	+9.10655	495.227	+15.8146	262.115	-0.1539	523.222	4	L	23	2508.2		2508.2	
	623.322		623.322		651.317	+5.55533	651.317	5	K	22	2395.11		2395.11	
	736.37		736.37		764.365		764.365	6	P	21	2267.02	-4.3585	2267.02	
	849.454		849.454		877.449		877.449	7	L	20	2153.97		2153.97	
	906.475		906.475		934.47		934.47	8	G	19	2040.89		2040.89	
	1066.51		1066.51		1094.5		1094.5	9	C	18	1983.86		1983.86	
	1123.53		1123.53		1151.52		1151.52	10	G	17	1823.83		912.421	+2.00876
	1180.55		1180.55		1208.54		1208.54	11	G	16	1766.81	+3.93007	1766.81	
	1294.59		1294.59		1322.59		1322.59	12	N	15	1709.79		855.399	+2.6262
	1351.61		1351.61		1379.61		1379.61	13	G	14	1595.75	+0.82084	1595.75	
	1464.7		1464.7		1492.69		1492.69	14	L	13	1538.73	-3.0617	1538.73	
	1563.77		1563.77		1591.76		1591.76	15	V	12	1425.64	-1.9888	1425.64	
-9.0973	855.921		1710.83		1738.83		1738.83	16	F	11	1326.57	-3.4249	1326.57	
	1797.87		1797.87		1825.86		1825.86	17	S	10	1179.51	+2.21055	1179.51	
	1868.9		1868.9		1896.9		1896.9	18	A	9	1092.47	-10.81	1092.47	
	1967.97		1967.97		1995.97		1995.97	19	V	8	1021.44	-1.0371	511.222	+3.07724
	2083		2083		2110.99		2110.99	20	D	7	922.368	-4.0764	461.688	+6.03138
-18.992	1099.02		2197.04		2225.04		2225.04	21	N	6	807.341	-1.8827	807.341	
	2312.07		2312.07		2340.06		2340.06	22	D	5	693.298	+0.90175	693.298	
	2472.1		2472.1		2500.09		2500.09	23	C	4	578.272	+1.27523	578.272	
	2587.13		2587.13		2615.12		2615.12	24	D	3	418.241		418.241	
	2715.22		2715.22		2743.22		2743.22	25	K	2	303.214	-0.151	303.214	
								26	R	1	175.119	+0.49689	175.119	

D

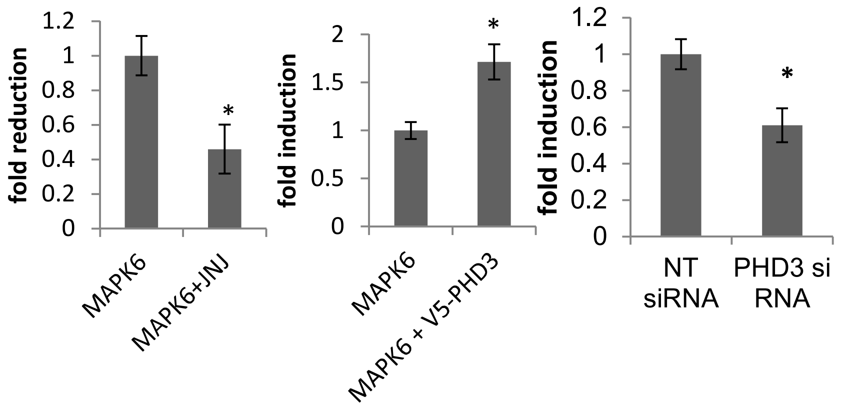

Figure S6

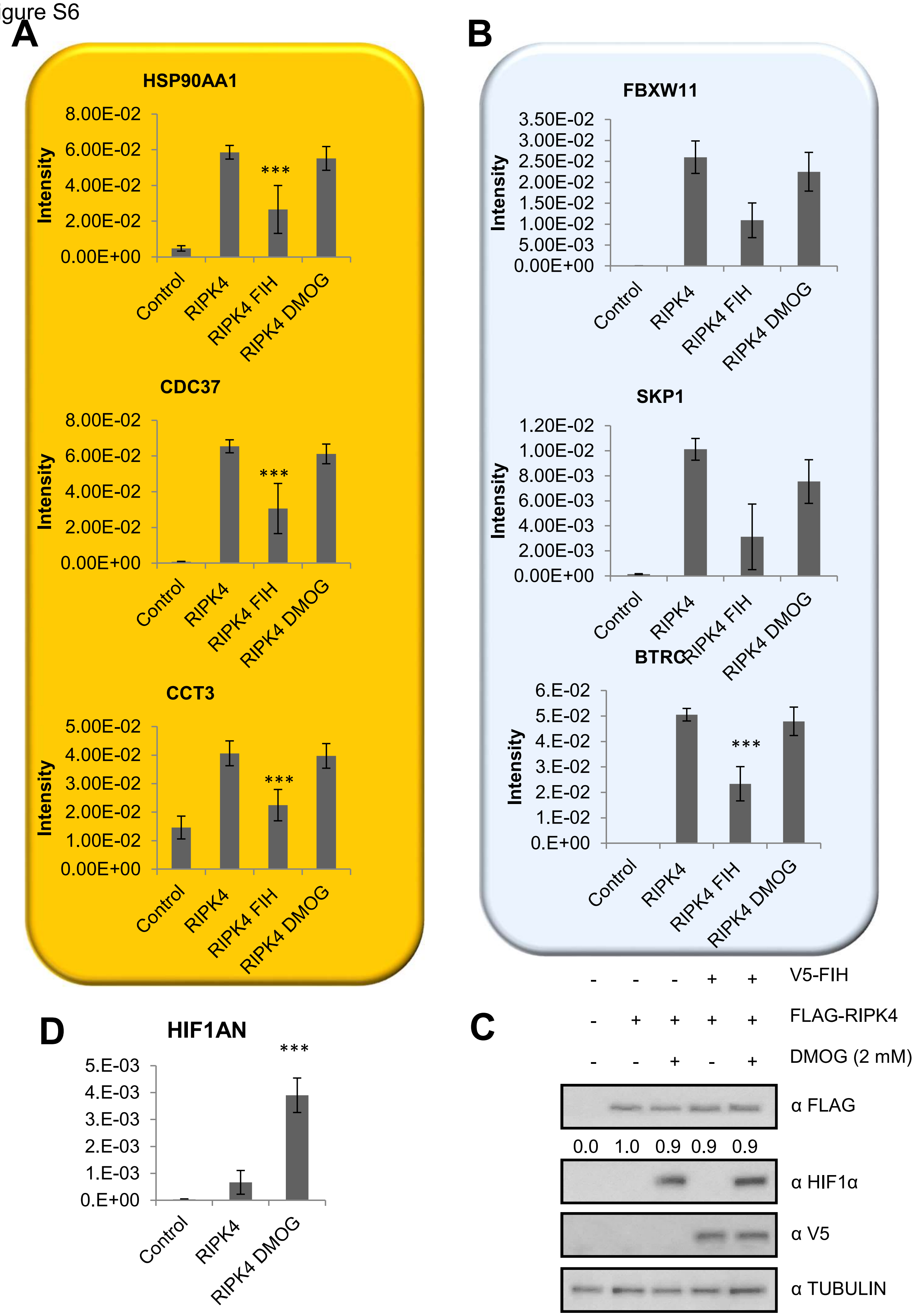

A
HSP90AA1
Intensity
8.00E-02
6.00E-02
4.00E-02
2.00E-02
0.00E+00
***
Control
RIPK4
RIPK4 FIH
RIPK4 DMOG
CDC37
CCT3
5.00E-02
4.00E-02
3.00E-02
2.00E-02
1.00E-02
B
FBXW11
3.50E-02
3.00E-02
2.50E-02
2.00E-02
1.50E-02
1.00E-02
5.00E-03
SKP1
1.20E-02
1.00E-02
8.00E-03
6.00E-03
4.00E-03
2.00E-03
BTRC
6.E-02
5.E-02
4.E-02
3.E-02
2.E-02
1.E-02
0.E+00
D
HIF1AN
5.E-03
4.E-03
3.E-03
2.E-03
1.E-03
0.E+00
C
V5-FIH
FLAG-RIPK4
DMOG (2 mM)
α FLAG
0.0 1.0 0.9 0.9 0.9
α HIF1α
α V5
α TUBULIN

Figure S7

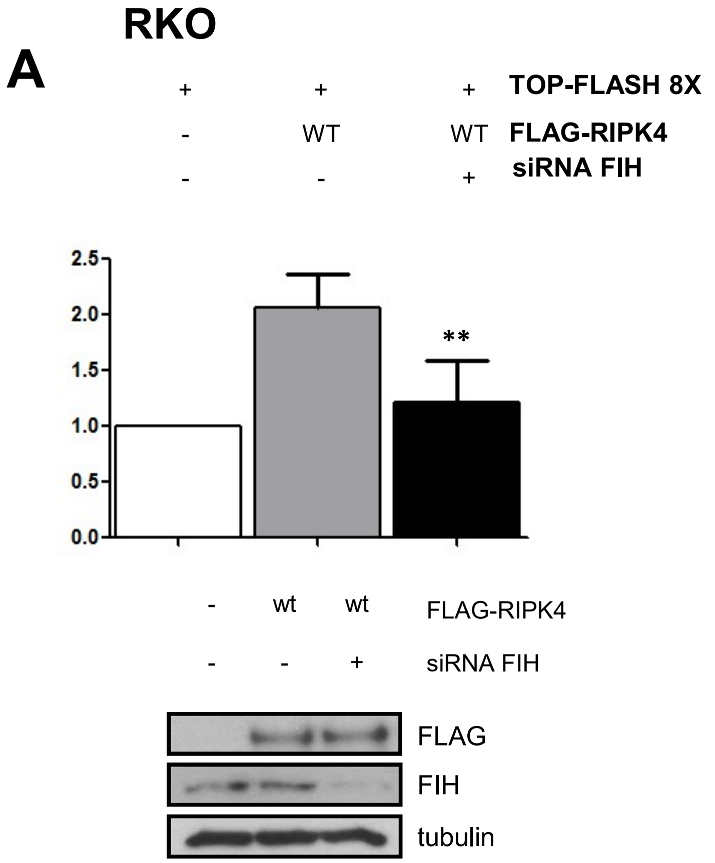

RKO
A
+ + + TOP-FLASH 8X
- WT WT FLAG-RIPK4
- - + siRNA FIH
2.5
2.0
1.5
1.0
0.5
0.0
**
- wt wt FLAG-RIPK4
- - + siRNA FIH
FLAG
FIH
tubulin

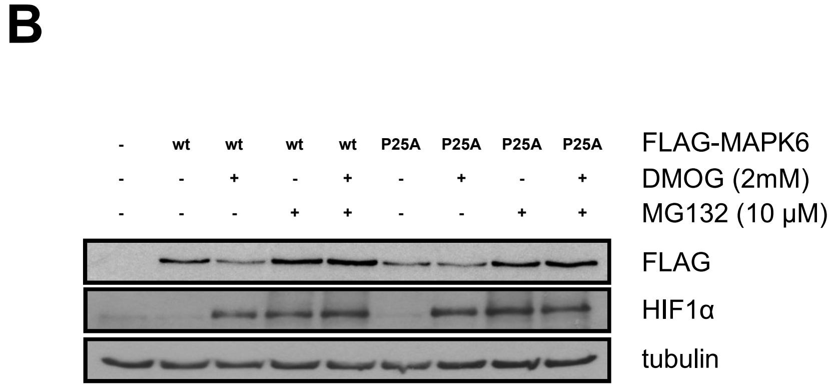

B
- wt wt wt wt P25A P25A P25A P25A FLAG-MAPK6
- - + - + - + - + DMOG (2mM)
- - - + + - - + + MG132 (10 µM)
FLAG
HIF1α
tubulin

Supplemental Figures

Figure S1. Related to Figure 1. Analysis of a steady-state model for hydroxylase-substrate interaction under inhibitor (DMOG) treatment. Dependence of total substrate-hydroxylase (Hdl-Sub) binding in response to gradual overexpression of the hydroxylase (Hdl) enzyme under varying dissociation constant (Kd) for reaction 1 (A) and reaction 2 (B). Kd1 and Kd2 are changed by changing either the forward (association) rate kf or the backward (dissociation) rate kr of the respective reactions. (C) FACS analysis of untransfected HEK293T cells. Cells were fixed, permeabilised and stained with PI. x-axis is detecting GFP, y-axis PI. (D) FACS analysis of HEK293T transfected with 1 µg of pmaxGFP. Cells were fixed, permeabilised and stained with PI. x-axis is detecting GFP, y-axis PI. (E) Comparison of C and D, x-axis GFP, y-axis counts. (F) FACS analysis of HEK293T transfected with 1, 0.7, 0.5 or 0.1 µg of pmaxGFP. Cells were fixed, permeabilised with PI. x-axis GFP, y-axis counts. (G) Linear representation of population median averages over µg of transfected pmaxGFP vector. (H) FIH is overexpressed 10-fold. HEK293T cells were transfected or not with V5-FIH. 24 hours post transfection the cells were lysed and proteins were separated by PAGE, electro-blotted and detected by the indicated antibodies. (I) PHD3 is overexpressed 30-fold. HEK293T cells were transfected or not with V5-PHD3. 24 hours post transfection the cells were lysed and proteins were separated by PAGE, electro-blotted and detected by the indicated antibodies.

Figure S2. Related to Figure 2. Volcano plot of how DMOG alter interaction dynamics of specific FIH and PHD3 interactors. (A) Scatter-plot of–log p-Value of the ttest comparing DMOG vs. untreated FIH-specific interaction (Table 1, Tab 2) over the log(2) of the ratio DMOG/untreated LFQ intensity. Selected interactors labelled with gene names. **(**B) Scatter-plot of–log p-Value of the ttest comparing DMOG vs. untreated PHD3-specific interaction (Table 2, Tab 2) over the log(2) of the ratio DMOG/untreated LFQ intensity. Selected interactors labelled with gene names.

Figure S3. Related to Figure 3. Identifying likely direct binder and substrates. (A) Visualisation of experimentally validated protein-protein interaction network of potential FIH substrates based on String DB. RIPK4 and TCEB1 and 2 highlighted. (B) LFQ intensities of the four most intensive interaction of the immediate TCEB1/2 network in the control/FIH immunoprecipitation untreated/DMOG treated. (C) Interaction dynamics of the four interactions seen in A. Ratio of LFQ intensity of DMOG/untreated FIH immunoprecipitation. (D) Fragmentation spectrum of the hydroxylation of N80 in endogenous ASB8. (E) Visualisation of experimentally validated protein-protein interaction network of potential PHD3 substrates based on String DB. MAPK6 is highlighted.

Figure S4. Related to Figure 4. Fragmentation spectra of hydroxylated RIPK4 peptides. (A) Normalised LFQ-intensities of RIPK4 binding to V5-FIH or a negative control. Bar graph representation of normalised LFQ-intensity values as obtained from V5-FIH immunoprecipitations. Error bars are SD n=3. (B) Normalised LFQ-intensities of MAPK6 binding to V5-PHD3 or a negative control. Bar graph representation of normalised LFQ-intensity values as obtained from V5-PHD3 immunoprecipitations. Error bars are SD n=3. (C) XIC of hydroxylated and their non-hydroxylated peptides respectively. Corresponding peaks are circled in red. Numbers (in red) represent the ratio of hydroxylated over the corresponding non-hydroxylated peptide. (D) HCD fragmentation spectra and y and b ion table of fragment ion which were mapped the corresponding RIPK4 peptides. (E) Fragment tables spectra in D and Fig. 4B. Mass is the m/z of the fragment ion in Da and Δppm represents the mass deviation in ppm of the measured fragment ion from the theoretical mass. Tables were extracted by the MaxQuant viewer, a program included in the MaxQuant suite.

Figure S5. Related to Figure 5. Fragmentation spectra of hydroxylated MAPK6 peptides. (A) XIC of the masses 726.09 (parent), 730.09 (parent P(ox)) and 729.84 (unrelated). The mono-isotopic P (hydroxylated) (red, eluting just before the unmodified peptide), mono-isotopic+1 unrelated (green, eluting just after the unmodified peptide) and their non-hydroxylated peptides respectively (blue). Corresponding peaks are underscored in red, green or blue. Numbers (in red) represent the ratio of hydroxylated over the corresponding non-hydroxylated peptide. (B) MS of 726.09 (parent), 730.09 (parent + 1 Oxygen) and 729.84 (unrelated). (C) Ion table of P25 hydroxylated peptide Fig. 4E. Mass is the m/z of the fragment ion in Da and Δppm represents the mass deviation in ppm of the measured fragment ion from the theoretical mass. The proline hydroxylation is localised by a y21, y21-H_2O, b5 and a b6-H_2O ion, resulting in a localisation probability of 0.999. Table was extracted by the MaxQuant viewer, a program included in the MaxQuant suite. (D) Bar-graph represents the normalised hydroxylation ratio of MAPK6 peptide YMDLKP(ox)LGCGGNGLVFSAVDNDCDKR in the presence/absence of JNJ or overexpressed V5-PHD3 or either non-targeting (NT) or PHD3 specific siRNA. . Error bars are SEM and n=3.

Figure S6. Related to Figure 5. RIPK4 and MAPK6 interaction screen. (A) Graphs showing RIPK4 interacting chaperone proteins which specifically change upon FIH over-expression. Bar-graphs representing LFQ-intensity values normalised to the RIPK4 input. Error bars are SD and n=6 (B) Graphs showing selected RIPK4 interacting SKP-complex proteins which specifically change upon FIH over-expression. Bar-graphs representing LFQ-intensity values normalised to the RIPK4 input. Error bars are standard deviation and n=6 (C) HEK293T cells were transfected with Flag-tagged RIPK4 with and without FIH and treated for 4 hours with DMOG 24 hours post transfection. The cells were lysed, proteins were separated by PAGE, electro-blotted and detected by the indicated antibodies. Western blot bands corresponding to RIPK4 were quantified and normalized against tubulin levels. (D) Graphs showing endogenous FIH interacting with exogenous Flag-RIPK4 in the presence/absence of DMOG. Bar-graphs representing LFQ-intensity values normalised to the RIPK4 input. Error bars are standard deviation and n=6

Figure S7. Related to Figures 5 and 7. Biological consequences of FIH or PHD3 hydroxylations. (A) RKO cells were transfected with FIH siRNA or non-targeting siRNA. 24 hours later re-transfected with vector, TCF/LEF luciferase reporter TOPFLASH-8, β-Gal, Flag-tagged RIPK4, the cells were lysed and the luciferase and β-Gal activity was measured or the protein were separated by PAGE, blotted and proteins were detected by the indicated antibodies. Bar graphs represent the luciferase activity normalised by β-Gal activity of three independent experiments with three biological replicates each (n=9). Error bars are standard deviation p-value < 0.05 =* <0,01=** (B) HEK293T cells were transfected with Flag-tagged MAPK6 or the P25A-mutant with and without V5-PHD3 and treated for 6 hours with DMOG or MG132 as indicated. The cells were lysed and proteins were separated by PAGE, electro-blotted and detected by the indicated antibodies.

Supplemental Tables

Table S1. Related to Figure 2. FIH immunoprecipitation data. HEK293T cells were transfected with V5-FIH or an empty vector and 48 hours post-transfection treated or not for four hours with DMOG. The cells were lysed and V5-FIH was immunoprecipitated and digested on-beads. Peptides were identified by mass spectrometry and quantified by LFQ by the MaxQuant software package. Contaminants and reverse data base hits were deleted. **(Sheet 1, raw data)** LFQ data as determined by MaxQuant. Column AD is the t-test of the untreated FIH (column M-R) vs. the negative control (column A-F). Column AE is the t-test of the DMOG-treated FIH (column S-X) vs. the negative control (column G-L). Column AG is the ratio of the averages of the untreated FIH/negative control intensities. Column AH is the ratio of the averages of the DMOG-treated FIH/negative control intensities. Highlighted in "orange" are proteins which are statistically enriched in the FIH samples under either condition (p<0.01 & ratio>2). **(Sheet 2, FIH interactome)** Subset of proteins statistically enriched in the FIH DMOG-treated sample vs. the negative control from Sheet 1 (p<0.01 & ratio>2). LFQ intensities are normalised to the FIH-input. Highlighted in "orange" are proteins which are statistically enriched in the FIH DMOG sample vs the FIH untreated sample. Protein expression changes as determined by whole cell LFQ expression proteomics. Experiments were run as biological triplicate, NaN indicates protein was not identified (X-AE). **(Sheet 3, FIH DMOG induced)** Normalised LFQ-intensities of potential FIH substrates. Column X to AE are the averages and standard deviation of the intensities and are used for the bar graph in Figs. 2, S2.

Table S2. Related to Figure 2. PHD3 immunoprecipitation data. HEK293T cells were transfected with V5-PHD3 or an empty vector and 48 hours post-transfection treated or not for four hours with DMOG. The cells were lysed and V5-PHD3 was immunoprecipitated and digested on-beads. Peptides were identified by mass spectrometry and quantified by LFQ by the MaxQuant software package. Contaminants and reverse data base hits were deleted. **(Sheet 1, raw data)** LFQ data as determined by MaxQuant. Column AD is the t-test of the untreated PHD3 (column M-R) vs. the negative control (column A-F). Column AE is the t-test of the DMOG-treated PHD3 (column S-X) vs. the negative control (column G-L). Column AG is the ratio of the averages of the untreated PHD3/negative control intensities. Column AH is the ratio of the averages of the DMOG-treated PHD3/negative control intensities. Highlighted in "orange" are proteins which are statistically enriched in the PHD3 samples under either condition (p<0.01 & ratio>2). **(Sheet 2, PHD3 interactome)** Subset of proteins statistically enriched in the PHD3 DMOG-treated sample vs. the negative control from Sheet 1 (p<0.01 & ratio>2). LFQ intensities are normalised to the PHD3-input. Highlighted in "orange" are proteins which are statistically enriched in the PHD3 DMOG sample vs the PHD3 untreated sample (Column AF p<0.01 & column AI ratio>1.5). Protein expression changes as determined by whole cell LFQ expression proteomics. Experiments were run as biological triplicate, NaN indicates protein was not identified (X-AE) **(Sheet 3, PHD3 DMOG induced)** Normalised LFQ-intensities of potential PHD3 substrates. Column X to AE are the averages and standard deviation of the intensities and are used for the bar graph in Figs. 2, S2.

Table S3. Related to Figure 5. RIPK4 interactome. HEK293T cells were transfected with Flag-RIPK4 or empty vector and an empty vector or V5-FIH and 48 hours post-transfection treated or not for four hours with DMOG. The cells were lysed and Flag-RIPK4 was immunoprecipitated and digested on-beads. Peptides were identified by mass spectrometry and quantified by LFQ by the MaxQuant software package and normalised to the RIPK4 input. Columns A-X are the LFQ intensities as determined by MaxQuant. A-F are the untreated negative control empty vector, G-L Flag-RIPK4 and FIH overexpressing and M-R untreated Flag-RIPK4 overexpressing and S-X are DMOG treated Flag-RIPK4 overexpressing samples. Columns Y-AA show the p-value of the t-test of the Flag-RIPK4 overexpressing samples vs the negative control and columns AB-AD show the ratios of the average LFQ intensities of the Flag-RIPK4 overexpressing samples over the negative control. Columns AE and AG show the p-value of the t-test of the Flag-RIPK4 overexpressing untreated or FIH overexpressing vs. the negative DMOG treated sample. AF and AH are the corresponding average ratios.

Table S4. Related to Figure 6. MAPK6 interactome. HEK293T cells were transfected with Flag-MAPK6 or empty vector and an empty vector or HA-PHD3 and 48 hours post-transfection treated or not for four hours with DMOG. The cells were lysed and Flag-MAPK6 was immunoprecipitated and digested on-beads. Peptides were identified by mass spectrometry and quantified by LFQ by the MaxQuant software package and normalised to the MAPK6 input. Columns A-X are the LFQ intensities as determined by MaxQuant. A-F are the untreated negative control empty vector, G-L untreated Flag-MAPK6 overexpressing, M-R are JNJ treated Flag-MAPK6 overexpressing and S-X Flag-MAPK6 and HA-PHD3 overexpressing samples. Columns Y-AA show the p-value of the t-test of the Flag-MAPK6 overexpressing samples vs the negative control and columns AB-AD show the ratios of the average LFQ intensities of the Flag-MAPK6 overexpressing samples over the negative control. Columns AE and AF show the p-value of the t-test of the Flag-MAPK6 overexpressing untreated or HA-PHD3 overexpressing vs. the negative JNJ treated sample. AG and AH are the corresponding average ratios.

Supplemental Experimental Procedures

Steady-state analysis of a DMOG-mediated substrate-trap model: Following the reaction steps leading to hydroxylation of HIF by PHD-2 described in Rose *et al.*(Rose et al., 2011), we derived a general schematic reactions diagram for the hydroxylation of a general substrate (Sub) by its respective hydroxylase (Hdl), as illustrated in Figure 1A. Under treatment of the hydroxylase inhibitors DMOG, which inhibits the hydroxylation step of the substrate, reaction 3 in the scheme is inhibited. Below we derived the expression for the total Substrate-Hydroxylase as a function of total abundances of the Substrate, Hydroxylate and Oxygen (O2).

The change of complexes Hdl-Sub and Hdl-Sub-O2 with time are given by the differential equations (1) and (2), where k1f, k1r and k2f, k2r are the association and dissociation rates for the Hydroxylase-Substrate binding and Hydroxylase-Substrate binding to Oxygen, respectively (reactions 1 and 2 in the scheme). k3 is the substrate hydroxylation catalytic rate but is assumed to be null under DMOG treatment.

$$d[\text{Hdl-Sub}]/dt = k1f*[\text{Sub}]*[\text{Hdl}] - k1r*[\text{Hdl-Sub}] - k2f*[\text{Hdl-Sub}]*[O2] \qquad (1)$$

$$d[\text{Hdl-Sub-O2}]/dt = k2f*[\text{Hdl-Sub}]*[O2] - k2r*[\text{Hdl-Sub-O2}] - k3*[\text{Hdl-Sub-O2}] \qquad (2)$$

At steady state, eqns (1) and (2) equal 0 and we obtain:

$$[\mathrm{Hdl\text{-}Sub}]= \frac{k1f}{k1r+k2f\,[O2]}[\mathrm{Hdl}]*[\mathrm{Sub}] \qquad (3)$$

$$[\mathrm{Hdl\text{-}Sub\text{-}O2}]= \frac{k1f}{k1r+k2f\,[O2]}\frac{k2f}{k2r}[\mathrm{Hdl}]*[\mathrm{Sub}] \qquad (4)$$

Moreover, the total abundances of Hdl and Sub (Hdl_{tot} and Sub_{tot}) are conserved and with (3) and (4) given as:

$$Hdl_{tot} = [\mathrm{Hdl}] + [\mathrm{Hdl\text{-}Sub}] + [\mathrm{Hdl\text{-}Sub\text{-}O2}]$$

$$= [\mathrm{Hdl}]\ (1+ \frac{k1f}{k1r+k2f\,[O2]}[\mathrm{Sub}] + \frac{k1f}{k1r+k2f\,[O2]}\frac{k2f}{k2r}[\mathrm{Sub}]) \qquad (5)$$

and

$$Sub_{tot} = [\mathrm{Sub}] + [\mathrm{Hdl\text{-}Sub}] + [\mathrm{Hdl\text{-}Sub\text{-}O2}]$$

$$= [\mathrm{Sub}]\ (1+ \frac{k1f}{k1r+k2f\,[O2]}[\mathrm{Hdl}] + \frac{k1f}{k1r+k2f\,[O2]}\frac{k2f}{k2r}[\mathrm{Hdl}]) \qquad (6)$$

Since the O2 concentration is abundant, we can assume $[O2] \approx [O2_{tot}]$ and solving equations (5), (6) we can obtain the steady-state form of [Hdl] and [Sub] as expression of Hdl_{tot} , Sub_{tot} and $O2_{tot.}$ Employing the Solve[ ] function in *Wolfram Mathematica 8*, these rather complex analytical expresions, are given below.

$$[Hdl] = \Big(-Subtot\,k1f\,k2r - k1r\,k2r - Subtot\,k1f\,k2f\,O2 - k2f\,k2r\,O2 + k1f\,k2r\,Hdltot + k1f\,k2f\,O2\,Hdltot - \sqrt{\big((Subtot\,k1f\,k2r + k1r\,k2r + Subtot\,k1f\,k2f\,O2 + k2f\,k2r\,O2 - k1f\,k2r\,Hdltot - k1f\,k2f\,O2\,Hdltot)^2 - 4\,(k1f\,k2r + k1f\,k2f\,O2)\,(-k1r\,k2r\,Hdltot - k2f\,k2r\,O2\,Hdltot)\big)}\Big) \Big/ (2\,(k1f\,k2r + k1f\,k2f\,O2))$$

$$[Sub] = Subtot - \frac{Subtot\,k1f\,k2r}{2\,(k1f\,k2r + k1f\,k2f\,O2)} - \frac{k1r\,k2r}{2\,(k1f\,k2r + k1f\,k2f\,O2)} - \frac{Subtot\,k1f\,k2f\,O2}{2\,(k1f\,k2r + k1f\,k2f\,O2)} - \frac{k2f\,k2r\,O2}{2\,(k1f\,k2r + k1f\,k2f\,O2)} - Hdltot + \frac{k1f\,k2r\,Hdltot}{2\,(k1f\,k2r + k1f\,k2f\,O2)} + \frac{k1f\,k2f\,O2\,Hdltot}{2\,(k1f\,k2r + k1f\,k2f\,O2)} - \Big(\sqrt{\big((Subtot\,k1f\,k2r + k1r\,k2r + Subtot\,k1f\,k2f\,O2 + k2f\,k2r\,O2 - k1f\,k2r\,Hdltot - k1f\,k2f\,O2\,Hdltot)^2 - 4\,(k1f\,k2r + k1f\,k2f\,O2)\,(-k1r\,k2r\,Hdltot - k2f\,k2r\,O2\,Hdltot)\big)}\Big) \Big/ (2\,(k1f\,k2r + k1f\,k2f\,O2))$$

Substituting these expressions into equations (3) and (4) we can obtain the total steady state substrate-hydroxylase complexes as a function of Hdl_{tot} , Sub_{tot}:

$$\Sigma[Hdl\text{-}Sub]\ complex = [Hdl\text{-}Sub] + [Hdl\text{-}Sub\text{-}O2] =$$

$$= [Hdl]*[Sub]\,\frac{k1f}{k1r + k2f\,[O2]}\,(1 + \frac{k2f}{k2r}) \qquad (7)$$

Using this function, we can analyse the dependence of the level of this complex on total substrate or hydroxylase abundances.

The dissociation rate for PHD2 and HIF1-CODD fragment binding has been reported (Fig.4 of Ehrismann *et al.* *(Ehrismann et al., 2007)*) to be about 0.186 s^{-1} and the association rate to be about 1.56 M^{-1} s^{-1}. Using nM and s as base units, we can assume a reference value for k1f and k1r to be:

$k1f = 1.56 * 10^{-6}\ nM^{-1}\ s^{-1}$

$k1r = 0.186\ s^{-1}$

On the other hand, the apparent Km for PHD2 with respect to Oxygen has also been reported to be about 250 µM (Hirsila *et al*.(Hirsila et al., 2005)). Since the catalytic rate (k_3) under DMOG is assumed to be zero, we can assume that this Km is equivalent to K_D of Hydroxylase-Oxygen binding (reaction 2). Taking

$k2f = 0.00001\ nM^{-1}\ s^{-1}$ (comparable to k1f) we then can compute

$$k2r = k2f * K_D = 250*10^3 * 0.00001 = 2.5\ s^{-1}$$

Putting these values into equation (7), we can obtain the dependency between the total Substrate-Hydroxylase complexes in response to increasing Hydroxylase (or Substrate) abundance (Fig. 1C, main text). We can see that over a wide dynamic range of the Hydroxylase spanning several orders of magnitude, a robust linear dependence is observed. This relationship persists even when we strongly varied the level of the substrate. Moreover, varying the binding affinity of reaction 1 and 2 over 2 orders of magnitude still maintains the observed linear dependence (Fig. E1).

Materials: All antibodies were from commercial sources: anti-FLAG M2 peroxidase was obtained from Sigma Aldrich (F4042, 1:1,000 dilution), anti-HIF1α was from BD Biosciences (610958 1:1,000 dilution), anti-tubulin and anti-ERK3 (MAPK6) were purchased from Santa Cruz (sc-8035/sc-365234, 1:1,000 dilution), anti-PHD3 was from Novus Biologicals (NB100-139, 1:1,000 dilution), anti-FIH was purchased from Abcam (1:1,000 dilution) and anti-V5 was obtained from Invitrogen (R96025, 1:5000 dilution). DMOG was obtained from Cayman Chemical (71210), MG132 was purchased from Sigma (M7449) and the PHD-specific inhibitor JNJ-42041935 was purchased from Merck Millipore (400093).

Plasmids and siRNAs: V5-PHD1-3 and V5-FIH was a gift from Cormac T. Taylor. FLAG-RIPK4 was a gift from Mathieu Bertrand, FLAG-MAPK4 was generated with the Gateway system. Flag–MAPK6-P25A mutant was made using the QuikChange kit (Stratagene) using following oligos CTAGGTATATGGACTTAAAAGCCTTGGGTTGTGGAGGCAATG (forward) CATTGCCTCCACAACCCAAGGCTTTTAAGTCCATATACCTAG (reverse). The non-targeting siRNA (siNT) and the siRNA targeting PHD3 (siPHD3) purchased from Dharmacon (ONTARGETplusSMARTpool), and the siRNA targeting FIH (siFIH) was produced by Eurogentec according to a previously reported sequence(Cockman et al., 2006).

ConA Sepharose fractionation: Cells were lysed in ice-cold lysis buffer (1% Triton-x100, 20 mM Tris-HCl (pH 7.5), 150 mM NaCl, 1 mM EDTA and 1 mM DTT), supplemented with protease (5 μg/ml leupeptin, 2,2 μg/ml aprotinin) and phosphatase (20 mM β-glycerophosphate) inhibitors. Lysates were cleared of debris by centrifugation at 20,000 x g for 10 min in a benchtop centrifuge. ConA Sepharose (GEHealthcare) were equilibrated with ConA buffer (20 mM Tris; 500 mM NaCl; 1 mM CaCl2; 1 mM MgCl2; pH 7.4). After sepharose equilibration, supernatants were incubated with ConA beads for 4h at 4ºC. The remaining supernatant was removed and diluted with Laemmli buffer, and analysed by Western blot.

Luciferase Reporter Assays: HEK293 or RKO cells were transfected with the M50 Super 8xTOPFlash reporter plasmid and the other indicated plasmids with LipofectAMINE 2000. After 16 hours cells were treated with DMSO or DMOG (2mM) for 3 hours Cells were washed once with phosphate-buffered saline and then lysed for 5 min at room temperature. The lysates were clarified by centrifugation at 14,000 rpm for 5 min and 20 μl of each lysate was used to measure luciferase reporter gene expression (luciferase assay kit, Promega). The luciferase activity was normalized to protein concentration or β-Gal. All experiments were performed in duplicate at least 3 times.

Substrate screening: HEK293T cells were plated (10^6 cells per culture dish) in 10 cm plates. The following day cells were transfected using Lipofectamine 2000 (according to the vendor's instructions) with 1 μg of empty vector (pCDNA 3.1) or V5 tagged PHD1-3 or FIH. After 16 hours cells were treated with DMSO or DMOG (2mM) for 3 hours. Cells were lysed in ice-cold lysis buffer (1% Triton-x100, 20 mM Tris-HCl (pH 7.5), 150 mM NaCl, 1 mM EDTA and 1 mM DTT), supplemented with protease (5 μg/ml leupeptin, 2,2 μg/ml aprotinin) and phosphatase (20 mM β-glycerophosphate) inhibitors. Lysates were cleared of debris by centrifugation at 20,000 x g for 10 min in a benchtop centrifuge. For immunoprecipitation anti-V5 beads (Sigma-Aldrich) or anti-Flag-M2 beads (Sigma Aldrich) were added to the cleared lysates and incubated at 4ºC under end-to-end rotation for 2 hours. Beads were washed three times with washing buffer (20 mM Tris-HCl (pH 7.5), 150 mM NaCl, 1 mM EDTA and 1 mM DTT). Subsequently, the samples were used for Mass Spectrometry analysis.

Tryptic on-bead digest: Following immunoprecipitation, samples were treated as published (Turriziani et al., 2014). Specifically, bound proteins were eluted in two steps. First, by using 60 μL of eluting buffer I [50 mM

Tris·HCl (pH 7.5), 2 M urea and 50 μg/mL trypsin (modified sequencing grade trypsin, Promega) and incubated while shaking at 28 °C for 30 min, and second, by adding twice 25 μL of elution buffer II [50 mM Tris·HCl (pH 7.5), 2 M urea and 1 mM DTT]. Both supernatants were combined and incubated overnight at room temperature. Samples were alkylated (20 μL Iodoacetamide, 5 mg/mL, 30 min in the dark). Then, the reaction was stopped with 1 μL 100% trifluoroacetic acid (TFA) and 100 μL of the sample was immediately loaded into equilibrated hand-made C18 StageTips containing Octadecyl C18 disks (Supelco). Samples were desalted by using two times 50 μL of 0.1% TFA and eluted with two times 25 μL of 50% AcN and 0.1% TFA solution. Final eluates were combined and concentrated until volume was reduced to 5 μL, using a CentriVap concentrator (Labconco). Samples were diluted to obtain a final volume of 12 μL by adding 0.1% TFA.

Expression proteomics: Biological triplicates of HEK293T cells treated or not for 3 h with DMOG were lysed with 1% SDS. Cell lysates were sonicated, assayed for protein content and boiled with 0.1M DTT. Sequential LysC and trypsin digests were performed as described (Farrell et al., 2014; Wisniewski and Rakus, 2014). Post-digest, 5 ug of LysC and 5 ug of trypitic peptides were analysed by LC-MS/MS.

Mass spectrometry: The tryptic peptides were analyzed on a Thermo Scientific Q-Exactive mass spectrometer connected to an Ultimate Ultra3000 chromatography system incorporating an autosampler. Five microliters of the resuspended tryptic peptides was loaded onto a homemade column (100-mm length, 75-mm inside diameter [i.d.]) packed with 1.9 μm RepreosilAQ C18 (Dr Maisch, Germany) and separated by an increasing acetonitrile gradient, using a 40-min (for interaction and hydroxylation experiments) or 240 min (for whole cell expression proteomics) reverse-phase gradient at a flow rate of 250 nl/min. The mass spectrometer was operated in positive ion mode with a capillary temperature of 220°C, with a potential of 2,000 V applied to the column. Data were acquired with the mass spectrometer operating in automatic data-dependent switching mode, selecting the 12 most intense ions prior to tandem MS (MS/MS) analysis.

The mass spectrometry proteomics data have been deposited to the ProteomeXchange Consortium via the PRIDE partner repository with the dataset identifier PXD001085 and the interaction data at IMEx accession IM-22750.

Data analysis: The mass spectrometry raw data was analysed by the MaxQuant 1.3 or 1.4 software packages using the pre-selected conditions for LFQ analysis. Specifically, MS/MS spectra were searched against the human Uniprot database with a mass accuracy of 6ppm and 20ppm (for MS and MS/MS). Carbamylation (c) was selected as fixed modification. Variable modifications were N-terminal acetylation (protein) and oxidation (M) for the interaction and expression screen, oxidation (MWYFKPHDN) for the hydroxylation screen. FDR was set to 0.01. LFQ and peak matching was selected and was limited to within a 30 s elution window with a mass accuracy of 6 ppm. The LFQ intensities were averaged across technical replicates and missing values were replaced by a constant (1). Normalisation was performed on the specific interactors by dividing the LFQ values by the average bait LFQ-intensity of the sample for each set of control and sample. Normalisation of hydroxylated peptides was performed by dividing the intensity of the modified by the matching unmodified peptide. The MAPK6 peptide containing the Pro(25) residue was further quantified by hand by comparing the XIC of the quadruply charged modified and unmodified peptides with a window of 0.02 Da.

Statistical Analysis: Technical replicates were averaged. Biological replicates and conditions were compared to each other by a two-tailed Student's t-test and by the ratio of the averages with the cut-off as indicated. N represents the number of biological replicates. Protein LFQ intensities are shown with error bars representing standard deviation, whereas values which rely on one measurement, such as hydroxylation intensities, are shown as standard error of mean (SEM). Statistical significance is indicated as not significant (NS) or p-value <0.05 (*). <0.01 (**) or <0.001(***).

In vitro hydroxylation assays: N-terminally biotinylated' synthetic peptides LLAKQPGVSV**N**AQTLDGRTPL and DLGSRYMDLK**P**LGCGGNGLVF (Selleckchem, USA) were incubated with lysates derived from HEK293T cells transiently transfected with V5-FIH, V5-PHD3 or a vector control as previously described (Yang et al., 2004). After the reaction was completed the peptides were eluted with biotin, desalted and analysed by LC-MS/MS. Flag-tagged RIPK4 and MAPK6 were IVT in rabbit reticulocytes using a T7 kit (Promega, USA) and purified with M2-Flag-agarose beads. The beads were subsequently incubated with lysates derived from HEK293T cells transiently transfected with V5-FIH, V5-PHD3, their inactive mutants H199A, H196A or a vector control as previously described (Yang et al., 2004). The beads were washed, digested with trypsin as described (Turriziani et al., 2014) and analysed by LC-MS/MS.

FACS analysis: HEK293T cells were transfected with maxGFP plasmid as indicated. After 24 hours, cells were collected by trypsinisation (0.05% Trypsin-EDTA, Gibco) and collected in growth medium (5ml). Cells were

centrifuged at 300 x g for 4 minutes and washed once in ice-cold PBS 1x. After resuspending the cells in 100 µl ice-cold PBS, 900 µl of ice-cold ethanol was added for fixation, and cells were stored at 4 C. Prior analysis cells were resuspended in 300 µl PBS containing Propidium Iodide (PI)(10 µg/ml) and RNAse A (100 µg/ml) and incubated in the dark at room temperature for 30 minutes. 50000 events (cells) for each sample were analysed with BD Accuri C6, using FL-1 for GFP (ex 488; em 530±30 nm) and FL-3 for PI (ex 488; em 670LP nm). Gating the population by forward and side scatter was performed in order to remove debris and doublets, resulting in the final "singlets in scatter" gating.

Supplemental References

Cockman, M.E., Lancaster, D.E., Stolze, I.P., Hewitson, K.S., McDonough, M.A., Coleman, M.L., Coles, C.H., Yu, X., Hay, R.T., Ley, S.C., *et al.* (2006). Posttranslational hydroxylation of ankyrin repeats in IkappaB proteins by the hypoxia-inducible factor (HIF) asparaginyl hydroxylase, factor inhibiting HIF (FIH). Proceedings of the National Academy of Sciences of the United States of America *103*, 14767-14772.

Ehrismann, D., Flashman, E., Genn, D.N., Mathioudakis, N., Hewitson, K.S., Ratcliffe, P.J., and Schofield, C.J. (2007). Studies on the activity of the hypoxia-inducible-factor hydroxylases using an oxygen consumption assay. The Biochemical journal *401*, 227-234.

Farrell, J., Kelly, C., Rauch, J., Kida, K., Garcia-Munoz, A., Monsefi, N., Turriziani, B., Doherty, C., Mehta, J.P., Matallanas, D., *et al.* (2014). HGF induces epithelial-to-mesenchymal transition by modulating the mammalian hippo/MST2 and ISG15 pathways. Journal of proteome research *13*, 2874-2886.

Hirsila, M., Koivunen, P., Xu, L., Seeley, T., Kivirikko, K.I., and Myllyharju, J. (2005). Effect of desferrioxamine and metals on the hydroxylases in the oxygen sensing pathway. FASEB journal : official publication of the Federation of American Societies for Experimental Biology *19*, 1308-1310.

Rose, N.R., McDonough, M.A., King, O.N., Kawamura, A., and Schofield, C.J. (2011). Inhibition of 2-oxoglutarate dependent oxygenases. Chemical Society reviews *40*, 4364-4397.

Turriziani, B., Garcia-Munoz, A., Pilkington, R., Raso, C., Kolch, W., and von Kriegsheim, A. (2014). On-beads digestion in conjunction with data-dependent mass spectrometry: a shortcut to quantitative and dynamic interaction proteomics. Biology *3*, 320-332.

Wisniewski, J.R., and Rakus, D. (2014). Multi-enzyme digestion FASP and the 'Total Protein Approach'-based absolute quantification of the Escherichia coli proteome. Journal of proteomics *109*, 322-331.

Yang, H., Ivan, M., Min, J.H., Kim, W.Y., and Kaelin, W.G., Jr. (2004). Analysis of von Hippel-Lindau hereditary cancer syndrome: implications of oxygen sensing. Methods in enzymology *381*, 320-335.
